# *Plasmodium falciparum* PfMyoF contains a Rab-like tail domain and associates with perinuclear membrane trafficking proteins

**DOI:** 10.1038/s42003-026-10192-1

**Published:** 2026-05-15

**Authors:** Alexander J. Holmes, Philip Ilani, Christopher Batters, Alison Kemp, Yaw Aniweh, Lucas N. Amenga-Etego, Gordon A. Awandare, Julian C. Rayner, Folma Buss

**Affiliations:** 1 Cambridge Institute for Medical Research, Department of Clinical Biochemistry, University of Cambridge, Cambridge Biomedical Campus, The Keith Peters Building, Cambridge, UK; 2 West African Centre for Cell Biology of Infectious Pathogens (WACCBIP), College of Basic and Applied Sciences, University of Ghana, Legon, Accra, Ghana

**Keywords:** Motor proteins, Malaria

## Abstract

Members of the myosin superfamily are found in all eukaryotes, including *Plasmodium falciparum*, the parasite that causes the majority of severe malaria. The *P. falciparum* genome encodes six myosins, but apart from PfMyoA and B, these remain largely uncharacterised. Here, we characterise PfMyoF, a class XXII myosin, using structural prediction, biochemical assays, and imaging. We show that PfMyoF is a plus-end-directed, processive motor with a long neck region with capacity to bind up to six copies of the calmodulin homologue PfCaM. The PfMyoF tail contains predicted WD40 and Rab-like domains that have not been identified in any other eukaryotic myosin class. Pull-down experiments identify PfMyoF interactions with trafficking proteins, including the vesicle marker PfRab18. Expansion and immunoelectron microscopy reveal that PfMyoF localises to a perinuclear membrane compartment and transient knockdown using the glmS ribozyme system impairs growth, potentially indicating an important function during asexual replication. Our findings define PfMyoF as the first myosin with a Rab-like domain and highlight its potential role in membrane trafficking pathways during the pathogenic blood stages of parasite development.

## Abstract (web summary)

PfMyoF is a unique, plus-end-directed *Plasmodium falciparum* myosin motor with WD40 and Rab-like tail domains. It binds PfCaM, associates with perinuclear membranes and trafficking proteins, and is likely important for blood-stage parasite growth.

## Introduction

Myosin motor proteins are ATP-dependent mechanoenzymes which generate mechanical force through movement along actin filaments, facilitating essential cellular processes such as intracellular transport, migration and cytokinesis^1^. Given the wide range of cellular processes involving myosin motors, several myosin classes have been successfully exploited as therapeutic targets for the treatment of a variety of human disease^2^. Although the myosin superfamily is a large and diverse group of proteins, they all share a conserved, tripartite domain organisation consisting of an N-terminal motor domain that binds actin and has ATPase activity, a neck region containing between one and six IQ motifs that bind light chains or calmodulin and, with the exception of *Apicomplexan* class XIV myosins, a highly variable C-terminal cargo-binding tail domain^3^.

While the human genome contains 40 myosin genes belonging to 12 classes, *Apicomplexan* parasites such as *Plasmodium falciparum*, the causative agent of the most severe cases of malaria, possess a limited and largely uncharacterised set of unconventional myosins that are distinct in sequence and structure from those of their human host. This evolutionary divergence offers a promising opportunity to exploit *P. falciparum* myosins as selective drug targets. Malaria remains the deadliest parasitic disease worldwide. In 2025, over 40% of the world’s population is at risk of infection, with the disease estimated to kill half a million people every year. According to the most recent data, malaria was responsible for at least 610, 000 deaths in 2024, 95% of which occurred in Africa^4,5^. While there are two WHO-recommended vaccines for *P. falciparum* with encouraging data, they do not offer complete protection and there are concerns that efficacy declines over time^6,7^. They do not yet therefore eliminate the need for antimalarial drugs as a central part of malaria control, and resistance to existing anti-malarial drugs is an increasing problem, underscoring the urgent need for new drug targets.

The six myosins identified in *P. falciparum* are PfMyoA, PfMyoB and PfMyoE of class XIV; PfMyoF of class XXII; and PfMyoJ and PfMyoK, which belong to a class VI-like group of myosins^8^. Among these, the essential myosin motor PfMyoA, and to some extent also PfMyoB, have been extensively characterised and shown to function in gliding motility and red blood cell invasion, a process essential for both parasite replication and malaria pathology^9,10^. Furthermore, PfMyoA has been successfully targeted using the small molecule inhibitor KNX-002, highlighting myosin motors as suitable candidates for anti-malarial drug development^11^.

PfMyoF, a myosin of class XXII, has been shown to function during the asexual blood stage of the *P. falciparum* life cycle, the stage responsible for the clinical symptoms and mortality associated with malaria^12^. Class XXII myosins are unique to *Apicomplexan* parasites, and despite their proposed essentiality, they remain poorly characterised in terms of motor properties, interaction partners, and functional roles within the parasite.

The most detailed studies of Myosin F in *Apicomplexa* have been in the related parasite *Toxoplasma gondii*, where TgMyoF functions in organelle positioning and dynamics. Disruption of TgMyoF function through expression of dominant-negative constructs or conditional knockdown impairs the dynamics and distribution of organelles, including dense granules (secretory organelles), the apicoplast (*Apicomplexan* metabolic plastids), and rhoptries (secretory organelles), as well as the Golgi and post-Golgi compartments positive for Rab5, Rab6, Rab7, Syn6 and DrpB^13–15^. In addition, the expression of dominant-negative TgMyoF constructs also caused centrosomal mislocalisation during cell division, indicating a role in maintaining centrosome positioning.

In *P. falciparum*, the first functional study of PfMyoF demonstrated its expression throughout the asexual blood cycle, particularly in the trophozoite and schizont stages^12^. Using a knock-sideways approach, the study showed that conditional mislocalisation of PfMyoF impaired haemoglobin uptake and parasite growth, implicating PfMyoF in endocytic processes essential for nutrient acquisition. A small fraction of PfMyoF was observed to colocalise with Kelch13-positive vesicles, structures associated with the endocytosis of host cytoplasm and potentially linking PfMyoF to artemisinin resistance; however, the C-terminal 2xFKBP-GFP-2xFKBP tag used in the study was found to affect PfMyoF function, leaving the precise localisation of endogenous PfMyoF unknown.

Genetic screens provide further evidence for the importance of class XXII myosin function in *Plasmodium* subspecies. In *Plasmodium berghei*, a rodent model of malaria, Wall et al. GFP-tagged all six myosins, revealing distinct patterns of expression and localisation across different life cycle stages^16^. Gene knockout experiments indicated that PbMyoA, PbMyoF and PbMyoK are likely essential, as deletion of these genes yielded no viable parasites. A recent high-saturation whole-genome piggyBac screen in *P. knowlesi* also identified PkMyoF as an essential protein for parasite viability^17^, whereas in *P. falciparum*, only PfMyoA was identified as essential in similar genome-wide screens^18^, although there is always a risk of false negatives using these approaches. Taken together, these findings suggest that PfMyoF may be essential for *P. falciparum* viability, but further targeted validation is needed.

While these studies have implicated PfMyoF in intracellular transport and parasite viability, its fundamental motor properties and cargo binding ability remain uncharacterised. To date, no studies have investigated the basic sequence and structural features of PfMyoF, including its oligomerisation status, directionality along actin filaments, domain architecture, or potential binding partners and endogenous localisation. Such insights are essential to understand the mechanistic basis of PfMyoF function and in assessing its potential as a therapeutic target.

Here, we address these knowledge gaps in PfMyoF using a combination of bioinformatics, structural modelling, in vitro expression and cell-based assays. We identify unique sequence and domain features of PfMyoF, including the discovery of a novel Rab-like domain within the tail. Using AlphaFold modelling, we further highlight that the PfMyoF lever arm contains six potential light chain binding sites for PfCaM, and suggest that PfMyoF is likely a dimer, supporting a role in processive cargo transport. Using a AlphaFold-based screen and a novel cell-based assay, we further suggest that PfMyoF is a plus-end-directed myosin. Finally, using a polyclonal antibody against the WD40 domain, we identify putative interaction partners and the cellular localisation of the endogenous protein at a perinuclear membrane compartment. Overall, this study combines computational and experimental approaches to define the core properties of PfMyoF, laying the groundwork for dissecting its role in *P. falciparum* biology.

## Results

### PfMyoF has a conserved motor domain, *Plasmodium*-specific insertions and a unique tail

**Fig. 1:**
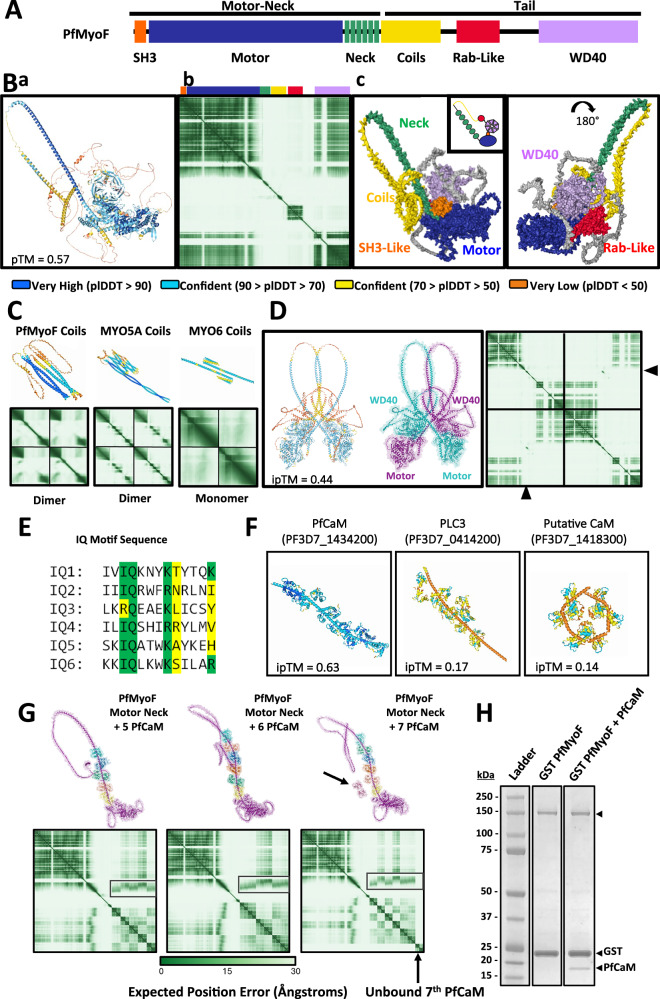
Predicted structural features and dimerisation potential of PfMyoF. **A** Schematic of the full-length PfMyoF, highlighting the N-terminal motor domain, neck with six IQ motifs, coiled-coil regions, Rab-like domain, and C-terminal WD40 domain. **B** AlphaFold-3-predicted structure based on the PfMyoF sequence. **Ba** PfMyoF coloured by pLDDT value. **Bb** PAE plot of PfMyoF. **Bc** PfMyoF coloured according to key domains. **C** AlphaFold-3-predicted structures of the coil domains of PfMyoF, HsMYO5A (positive control), and HsMYO6 (negative control). Structures are coloured by pLDDT with corresponding PAE plots presented below. **D** AlphaFold-3 prediction of PfMyoF dimers shows coiled-coil interaction with a backfolded architecture. Black arrowheads point to the coiled-coil interaction within the PAE plot. **E** Sequence alignment showing the six putative IQ motifs within PfMyoF neck domain. Green sequences highlight the canonical IQ motif amino acids, and yellow sequences show degenerate sequences. **F** AlphaFold-3 prediction of putative light chains PfCaM, PLC3, and PF3D7_1418300 shows that only PfCaM binds to all six IQ motifs in the PfMyoF neck with high ipTM confidence (0.63). **G** AlphaFold-3 modelling predicts binding of up to six PfCaM molecules along the PfMyoF neck domain (purple), ending at the beginning of the coil domain. Additional PfCaM proteins remain unbound (indicated on the PAE plot by a black arrow). **H** SDS-PAGE gel highlighting co-purification of 6His-PfCaM with GST-PfMyoF motor neck from Sf9 cells, confirming its interaction with PfMyoF.

To gain insight into the structural organisation of PfMyoF, we used AlphaFold to generate a full-length structural model. The predicted PfMyoF structure follows a canonical myosin organisation with the motor domain at the N-terminus, followed by a long neck domain, an extended coiled-coil region, and two unique C-terminal tail domains: a previously unannotated Rab-like domain, and finally a WD40 β-propeller (Fig. 1A, B). In this AlphaFold model, PfMyoF adopts a compact, backfolded structure, suggestive of an autoinhibited conformation (Fig. 1Ba–c) that resembles TgMyoF but is distinct from other classes of *Apicomplexan* myosins (Fig. S1). The model has a moderate pTM score of 0.57, indicative of a reasonably confident prediction, with higher local confidence in the motor and individual tail domains highlighted by darker green in the Predicted Aligned Error (PAE) plot (Fig. 1Bb).

The motor domain of PfMyoF shares core architectural features with other myosin classes, including the essential conserved ATP-binding P-loop (GESGAGKT), the actin-binding interface, and a preserved cleft architecture (Figs. 1B and S2A). Other key myosin features, including the TEDS site, which is a regulatory phosphorylation site common to unconventional myosins, is also conserved. PfMyoF, like all *P. falciparum* myosins, contains an N-terminal SH3-like domain (Fig. S2A). In human myosins MYO5A and MYO6, this domain interacts with the tail to stabilise the folded autoinhibited conformation^19,20^. Unlike the well-characterised PfMyoA, which contains a phospho-regulated S19 residue preceding its SH3-like domain, PfMyoF lacks this extension and phosphorylation motif^21^.

The *Plasmodium* genome is highly AT-rich, and the PfMyoF sequence reflects this bias, with an overall AT content of ~75% (in contrast to human myosins like MYO5A with an AT content of 55%). This compositional skew likely contributes to the presence of large, asparagine-rich insertions not observed in myosins from other organisms such as *Toxoplasma* TgMyoF (Figs. 1Ba, c and S2A–C). These insertions are predicted to be unstructured and may influence folding, solubility, or regulation.

Together, these findings indicate that PfMyoF retains a structurally conserved core motor domain but has evolved *Plasmodium*-specific insertions and unique tail domains, which may be central to its parasite-specific functions and represent potential therapeutic targets.

### AlphaFold-3 prediction indicates that PfMyoF has the potential to form dimers

Myosins can be broadly classified based on their oligomeric state into monomeric (single-headed) or dimeric (double-headed) motors, such as myosins of class V. Dimerisation, which enables hand-over-hand, processive movement along actin filaments, typically occurs via coiled-coil formation in alpha-helical regions within their tail domains. The coiled-coil prediction software MARCOIL identifies two coiled-coil regions in the PfMyoF tail^22^. To assess their dimerisation potential, we used AlphaFold-3, which accurately distinguishes between the dimeric MYO5A and the monomeric MYO6: MYO5A is predicted to form a coiled-coil dimer, whereas MYO6 contains a single alpha helix (SAH domain) that does not dimerise (Fig. 1C)^23^. Similarly to MYO5A, AlphaFold-3 predicted that two copies of PfMyoF can form a dimer mediated by coiled-coil interactions (Fig. 1C). The contribution of these coiled-coil regions to PfMyoF or MYO5A dimerisation is highlighted by the PAE plots (Fig. 1C). Furthermore, the full-length dimeric PfMyoF is predicted to adopt an autoinhibited conformation with the tail domains back-folding to interact with the motor domains, as observed for MYO5A (Fig. 1D)^24^. However, we emphasise that these AlphaFold-based structural predictions require future experimental confirmation to validate both the PfMyoF dimerisation potential and the proposed autoinhibited conformation.

### PfMyoF is predicted to contain six light chain binding sites and demonstrates the capacity to bind PfCaM

Next, we focused on identifying the PfMyoF light chains, which are critical for activation and regulation of motor properties of all myosins. The neck domain, together with the converter region, forms the swinging lever arm that drives myosin movement upon nucleotide exchange^25^. Calmodulin-like light chains bind to IQ motifs within the neck, stabilising and regulating the swinging lever arm motion. To date, only the light chains of PfMyoA and PfMyoB from *P. falciparum* have been identified. PfMyoA binds the MTIP (PF3D7_1246400) and ELC (PF3D7_1017500) light chains^21,26,27^, whereas PfMyoB binds to MLC-B (PF3D7_1118700) and potentially other light chains^10^. While many human unconventional myosins complex with calmodulin, neither PfMyoA or PfMyoB appear to bind the *P. falciparum* calmodulin homologue (PfCaM).

Sequence analysis of PfMyoF identified six degenerate IQ motifs broadly matching the consensus sequence ‘[I,L,V]QxxxRGxxx[R,K]’, suggesting the presence of six putative light chain binding sites (Fig. 1E). In addition to PfCaM, which shows 89% sequence conservation with human CaM, nine other calmodulin-like proteins were identified in the *P. falciparum* genome based on sequence identity, and all were screened for their ability to bind to the PfMyoF neck domain using AlphaFold-3 (Fig. 1F). Modelling with six PfCaM copies yielded an ipTM score of 0.63, which exceeds the 0.6 threshold for confident interaction, supporting a plausible linear lever arm structure (Fig. 1F, left panel). In contrast, all other CaM-like proteins gave lower ipTM scores (ranging from 0.13 to 0.36) indicating weaker or unlikely interactions (Fig. 1F, middle and right panel).

Myosins with six IQ motifs, such as human MYO5A, typically bind multiple copies of a single light chain. AlphaFold-3 modelling of PfCaM binding to the neck domain of PfMyoF showed that PfCaM could bind sequentially to all six IQ motifs, saturating at six molecules with no additional binding (Fig. 1G).

The AlphaFold-3 prediction was verified experimentally by co-expressing GFP-tagged PfMyoF motor-neck alongside 6xHis-PfCaM in Sf9 insect cells (Fig. 1H). Small quantities of soluble GFP-PfMyoF motor-neck could be purified using GST-GFP nanobody bound to Glutathione Sepharose and analysed by SDS-PAGE. In Sf9 cells expressing only PfMyoF, no band is present at the expected kDa of a light chain such as PfCaM. However, in cells co-expressing PfMyoF and PfCaM, an additional 18 kDa band is present at the predicted size for 6His-PfCaM (Fig. 1H). This co-purification of PfCaM with PfMyoF provides direct evidence that PfCaM can function as a PfMyoF light chain.

### The unique Rab-like domain of PfMyoF

The functional differences between classes of unconventional myosins are determined by their unique tail domains, which contain distinct motifs and binding sites. As such, the tail domain determines both the cellular localisation and binding partners of the myosin, allowing for anchoring, scaffolding, or transportation functions^28,29^.

**Fig. 2:**
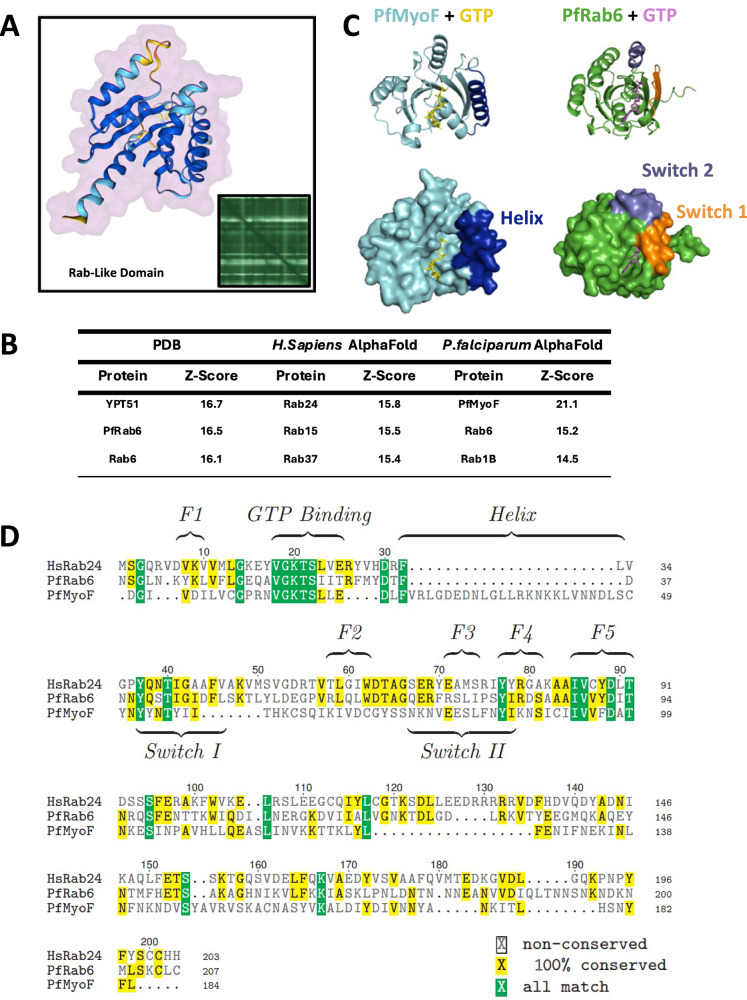
Identification and structural characterisation of the PfMyoF Rab-like domain. **A** AlphaFold-3 predicted structure of PfMyoF Rab-Like domain. **B** DALI structural homology search identifies significant similarity to *Plasmodium* and human Rab GTPases. A table detailing the top hits from a DALI server search of the protein data bank (PDB) and AlphaFold servers for proteins with a similar structure to the PfMyoF Rab-Like domain. *Z*-scores, a measure of significant similarities, are shown. **C** AlphaFold-3 predicted structure of PfMyoF Rab-Like domain and PfRab6 bound to GTP. The additional helix in PfMyoF appears to disrupt the GTP binding pocket and reposition the key Switch I and Switch II sites essential for GTP hydrolysis. **D** Sequence alignment of PfMyoF Rab-Like domain, PfRab6 and HsRab24 reveals a conserved GTP-binding site but divergence in Switch I and II regions. Residues are coloured by conservation, and regions of interest are labelled.

The PfMyoF tail domain contains at its C-terminus a WD40 domain, as previously described^8^. Structural predictions revealed a second previously undefined domain between the coiled-coil and WD40 domains, which shows a strong similarity to Rab proteins of the Ras family (Figs. 2A and S3). Rab proteins are activated by GTP binding, enabling the recruitment of trafficking machinery to vesicles and organellar membranes^30^. A Rab-like domain has not been identified in any myosin tail to date, raising questions about its function and potential for GTPase activity within the PfMyoF tail.

Sequence homology searches for this PfMyoF Rab-like domain using BLAST^31^ identified the 11 known *P. falciparum* Rab proteins as the top hits. The Golgi-localised PfRab6 ranked highest in terms of sequence (based on BLAST) and structural homology (based on DALI) to the PfMyoF Rab domain. Structural homology modelling using the DALI server further compared the AlphaFold-predicted structure of this domain to known protein structures in the protein data bank (PDB) and other AlphaFold-predicted protein structures^32^. All the top hits (ordered by *Z*-score, a measure of significant similarity) from both comparisons indicated strong homology to Rab family proteins (Fig. 2B).

Overlay of the AlphaFold-3-predicted structures of PfRab6 and PfMyoF Rab-like domains reveals high structural similarity. PfMyoF possesses an additional α-helix that is conserved in a number of *Apicomplexan* Myosin F proteins (Fig. 2C, helix highlighted in dark blue). We next compared the sequences of the PfMyoF Rab-like domain with those of PfRab6 and human Rab24. These Rab proteins were selected based on combined sequence- and structure-based homology searches, as PfRab6 and human Rab24 are the highest-ranking parasite and human hits, compared to the PfMyoF Rab-like domain in both BLAST and DALI analyses. This sequence alignment shows conservation of the GTP-binding site in PfMyoF, but poor conservation of the Switch I and Switch II regions required for γ-phosphate binding and hydrolysis, suggesting that the Rab-like domain of PfMyoF is a pseudodomain that may not possess intrinsic GTPase activity (Fig. 2D). Structural alignment indicates that the additional α-helix results in the rearrangement and opening of the GTP binding cleft, and displacement of the switch regions away from the γ-phosphate of GTP (Fig. 2C).

### The WD40 domain of PfMyoF

**Fig. 3:**
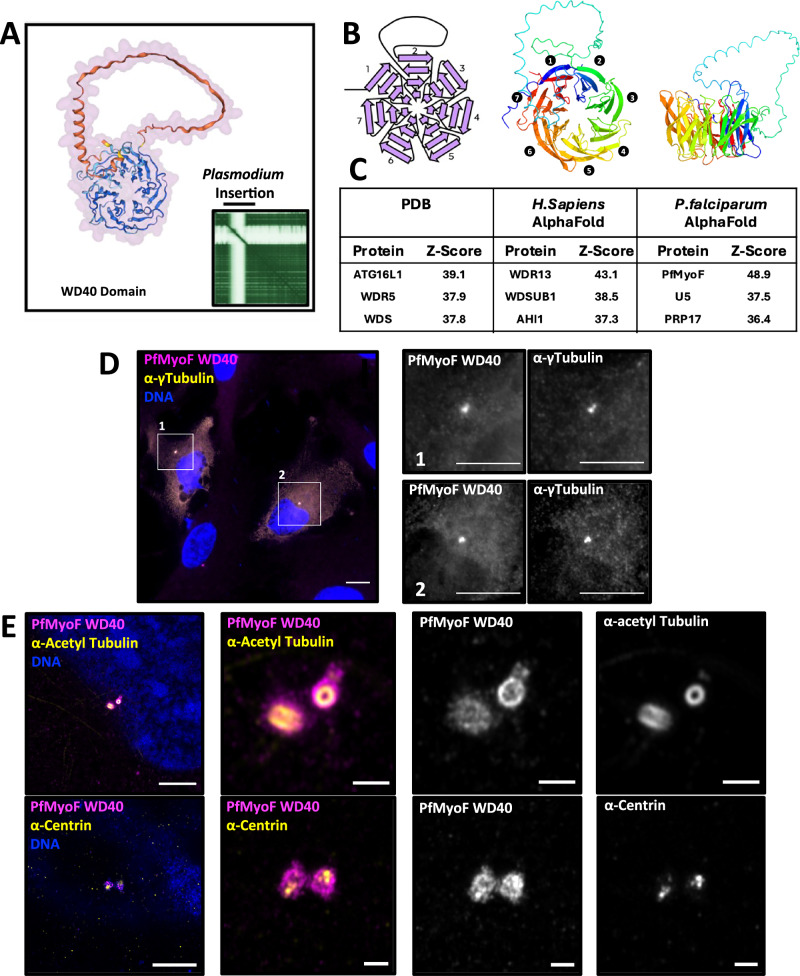
Characterisation and centrosomal localisation of the PfMyoF WD40 domain in mammalian cells. **A** AlphaFold-3 predicted structure of PfMyoF WD40 domain, forming a 7-bladed β-propeller structure with a *Plasmodium*-specific insertion. **B** A schematic of PfMyoF WD40 (left) highlighting the 7-blade structure as well as an N-terminal to C-terminal rainbow colour scheme in top view (middle) and side view (right). **C** A table detailing the top hits from a DALI server search of the protein data bank (PDB) and AlphaFold servers for proteins with a similar structure to the PfMyoF WD40 domain. *Z*-scores, a measure of significant similarities, are shown. **D** Confocal microscopy of GFP-tagged PfMyoF WD40 expressed in RPE cells stained with anti-GFP (magenta), anti-γTubulin (yellow) and Hoechst (blue). Scale bars represent 10 µm. **E** iU-ExM of RPE cells transiently expressing GFP-tagged PfMyoF WD40 domain and stained with anti-GFP (magenta), Hoechst (blue) and either anti-Centrin or anti-Acetyl-αTubulin (yellow). Scale bars represent 10 µm, or 2 µm for zooms.

The second of the two unique domains within the PfMyoF tail region is a WD40 domain (Fig. 3A). WD40 domains consist of repeating WDR motifs that form the ‘blades’ of a rounded β-propeller and typically function by assembling multi-protein scaffolds on this propellor^33^. Sequence analysis and AlphaFold prediction indicate that the PfMyoF WD40 domain contains 7 blades, including a large 58-residue unstructured insertion between blades 1 and 2, unique to *Plasmodium* species (Fig. 3B).

To further classify and characterise the PfMyoF WD40 domain, sequence and structural homology searches were performed using BLAST and DALI servers (Fig. 3C). DALI analysis identified similar structures within diverse proteins, including human WDR5 (histone-related), WDR13 (transcription-related), and AHI1 (Jouberin), which interacts with GTPases such as Rab8A to mediate the recruitment of Rabs and membrane trafficking machinery to the basal body of primary cilia. In the *P. falciparum* AlphaFold database, top hits included PRP17, among other spliceosome components. This functional diversity between homologous proteins warrants further investigation into the evolutionary and functional origins of the WD40 domain. While WD40 domains have not been observed in higher eukaryotic myosin tail domains, *Apicomplexan* parasites have evolved multiple myosin classes that contain them, including PfMyoF and PfMyoJ (harbouring a WD55-like version) in *P. falciparum*, as well as TgMyoF, TgMyoH, TgMyoJ, and TgMyoL in *T. gondii* (Fig. S1). These WD40 domains show considerable sequence and structural divergence, suggesting independent adaptation and distinct functions across classes.

Previous studies in *T. gondii* found that the TgMyoF WD40 domain binds to microtubules and that TgMyoF associates transiently with the centrosome during cell division^13,34,35^. As many WD40 domain-containing proteins have been shown to localise to the centrosome in several species, we transiently transfected the GFP-tagged PfMyoF WD40 domain into human retinal pigment epithelial (RPE) cells and visualised its localisation. Our results show that GFP-tagged PfMyoF WD40 strongly colocalised with γ-tubulin at a single punctum resembling the centrosome (Fig. 3D). Iterative Ultrastructural Expansion microscopy (iU-ExM) showed that the PfMyoF WD40 domain localises to the area surrounding acetylated-α-tubulin and centrin, which form a core component of the centrosome (Fig. 3E)^36^. This observation suggests potential binding of the WD40 domain to the outer microtubules or components of the pericentrosomal material.

### PfMyoF is a plus-end-directed myosin motor

Myosin motors can either walk towards the plus or the minus end of actin filaments, and this directionality is crucial for their function in organelle positioning, cargo transport, and actin organisation. For example, reversing the direction of class VI myosin (MYO6) motility so that it adopts a plus-end directed (MYO6+) phenotype results in a dramatic relocalisation of APPL1-positive signalling endosomes in HeLa cells^37^. Although *P. falciparum* myosins PfMyoJ and PfMyoK have been categorised as ‘class VI-like’, suggesting they may be minus-end directed actin motors, the directionality of PfMyoF remains unknown^8^.

A crucial determinant of minus-end-directed motility is Insert 2, located at the C-terminus of the converter region within MYO6, which reorients the lever arm swing^38^. Multiple sequence alignments reveal that PfMyoF lacks any Insert 2-like sequence, whereas both PfMyoJ and PfMyoK contain an insertion at the C-terminal end of their converter domain (Fig. S4). The PfMyoJ insert shows strong homology with Insert 2 of MYO6 (28% identical and 47% similarity), higher than that of PfMyoK (12% identical and 35% similarity) (Fig. S4).

**Fig. 4:**
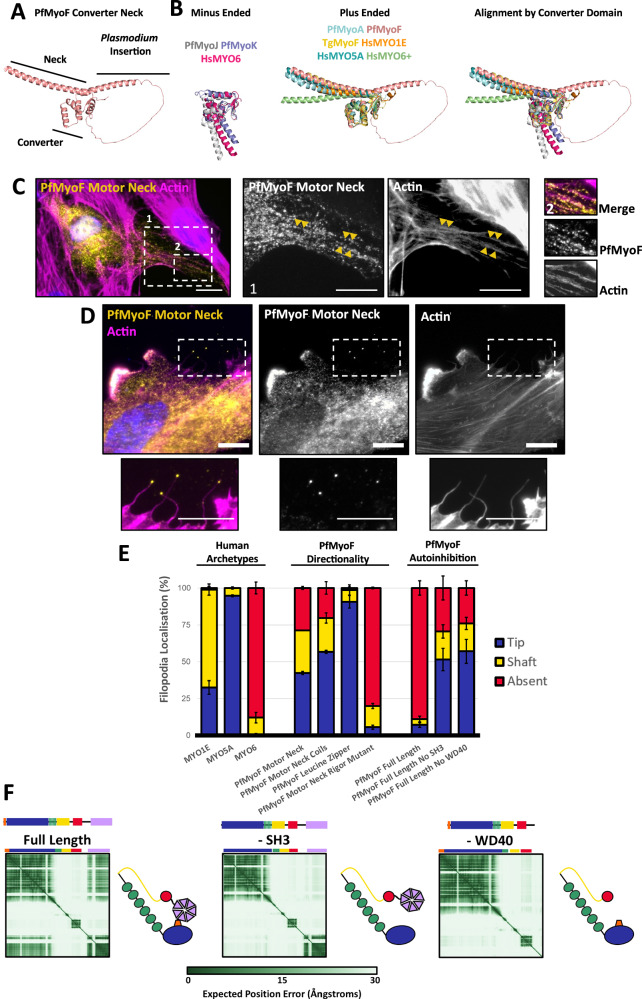
PfMyoF is a plus-end directed myosin motor. **A** AlphaFold-3 prediction of the PfMyoF converter-neck region highlights a larger insertion within the converter domain. **B** Alignment of HsMYO6 with PfMyoJ and K (left panel) and alignment of PfMyoA, PfMyoF, TgMyoF, HsMYO1E, HsMYO5A, HsMYO6+ by their converter domain (middle panel). In the right panel, the overlay of both minus-ended (left panel) and plus-ended subgroups (middle panel) is shown. **C** PfMyoF aligns along actin filament bundles and accumulates in membrane ruffles in RPE cells transiently transfected with GFP-PfMyoF motor neck; GFP (yellow), Phalloidin (magenta) and Hoechst (blue). Scale bars represent 10 µm. **D** PfMyoF localises to filopodia tips. RPE cells were transfected with GFP-tagged PfMyoF motor neck and mCherry-tagged MYO6+ and stained with anti-GFP (yellow), Phalloidin (magenta) and Hoechst (blue). Scale bars represent 10 µm. **E** Quantification of filopodia tip localisation as a measure of their directionality across different human myosin classes and PfMyoF constructs. RPE cells were co-transfected with various GFP-tagged myosins alongside mCherry-tagged MYO6+. Per construct, 100 filopodia were counted in 3 independent experiments (*n* = 3) and classified as either ‘tip’, ‘shaft’ or ‘absent’ localisation. These experiments were performed blinded. Error bars indicate SEM. **F** AlphaFold-3 predicts autoinhibited backfolding of PfMyoF via interaction between SH3-like and WD40 domains. Shown are the PAE plots and corresponding protein models of PfMyoF full-length, delta SH3-like or delta-WD40, which suggest that autoinhibition may be mediated through the SH3-like and WD40 domains.

Although PfMyoF lacks Insert 2 adjacent to the converter domain, it contains a unique large insertion *within* the converter domain (Fig. 4A). To investigate whether this PfMyoF converter insertion potentially alters motor directionality along actin filaments, we developed an AlphaFold-3-based approach to examine the impact of sequence insertions on lever-arm angle. Alignment of the converter-neck regions from all human unconventional myosins correctly distinguished plus-ended from minus-ended motors (Fig. S4). Applying this method to PfMyoA, PfMyoF, PfMyoJ, and PfMyoK along with TgMyoF (which lacks the PfMyoF converter insert), revealed that PfMyoA, PfMyoF and TgMyoF converter-necks align with plus-ended myosins, whereas PfMyoJ and PfMyoK align with minus-ended motors (Fig. 4B). A MYO6+ chimera lacking Insert 2, aligned with plus-end motors, consistent with experimental data^37^ (Fig. 4B). Together, these sequence and structural predictions suggest that PfMyoF is a plus-end-directed motor, while PfMyoJ and PfMyoK exhibit characteristics of minus-ended myosins.

To experimentally validate these AlphaFold predictions in a biological system, we expressed a GFP-tagged PfMyoF motor-neck construct in RPE cells, where it localised along actin filaments and accumulated in actin-rich membrane ruffles, indicating correct folding and actin binding in mammalian cells (Fig. 4C). To set up a directionality assay, we co-expressed a MYO6+ chimera to induce long filopodia, in which the plus ends of actin filaments point toward the filopodia tip. Different myosin classes localise to distinct regions within filopodia according to their intrinsic motor properties (Fig. S5). Dimeric and processive plus-ended myosins, such as MYO5A, accumulate at the tips of the filopodia, while monomeric plus-ended myosins with membrane-binding capacity, such as those of class I, are evenly distributed along the filopodial shaft. In contrast, minus-ended myosins like MYO6 are excluded from filopodia and accumulate at the base. This assay has been previously validated with *C. elegans* myosins of known directionality^39^.

In RPE cells, GFP-tagged PfMyoF motor-neck localises to filopodia tips, a hallmark of plus-end directed, processive motors (Fig. 4D). Inclusion of the PfMyoF coiled-coil region increased tip localisation from 42% to 56%, likely due to dimer formation via the coiled-coil domain, which enhances processivity (Fig. 4E). Insertion of a heterologous C-terminal leucine zipper to artificially induces dimer formation further increased PfMyoF tip localisation to 90%, confirming the importance of dimerisation (Fig. 4E). In contrast, mutation of the PfMyoF motor by insertion of an ATPase-deficient ‘rigour’ mutation (K_415_R) previously shown to inhibit MYO6 activity, abolished PfMyoF tip localisation (5%) (Fig. 4E).

In contrast to the GFP-PfMyoF motor-neck construct, the full-length PfMyoF showed minimal tip localisation (7%) possibly due to misfolding, targeting of the tail domain to other cellular sites, or autoinhibition (Fig. 4E). AlphaFold-3 predicts both monomeric and dimeric full-length PfMyoF to adopt a backfolded, autoinhibited conformation mediated by interactions between the N-terminal SH3-like domain and the WD40 domain (Fig. 4F). Deleting either the SH3-like or WD40 domains restored PfMyoF tip localisation to 51% and 57%, respectively, matching the PfMyoF motor-neck-coiled-coil construct (Fig. 4E).

Overall, these results validate our cell-based assay for testing myosin directionality and suggest that PfMyoF is most likely a plus-end-directed motor, with dimerisation and autoinhibition potentially contributing to the regulation of its activity and localisation in the parasite.

### Characterisation of PfMyoF interactome via co-immunoprecipitation reveals a link to membrane trafficking proteins

**Fig. 5:**
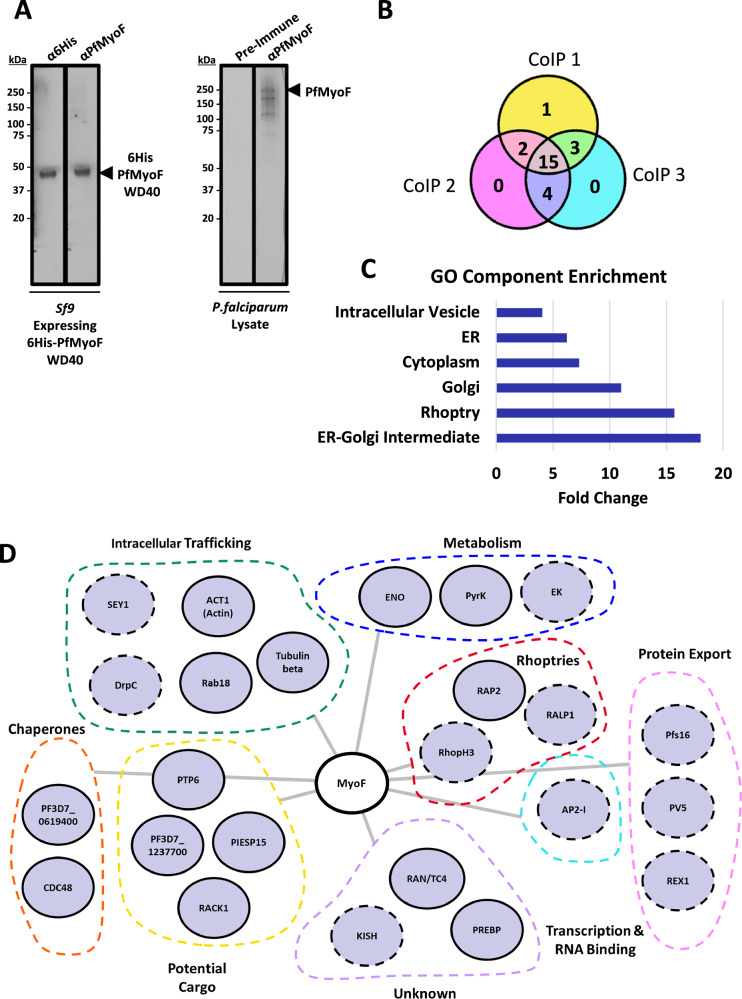
PfMyoF interactome identified by native co-immunoprecipitations. **A** Immunoblot validation of α-PfMyoF antibody against recombinant WD40 fragment (left panels) and endogenous PfMyoF from parasite lysates (right panels). Sf9 cell lysate expressing 6His-WD40 domain (left panels) was probed with anti-6His (left lane) or anti-PfMyoF antibodies (right lane). *P. falciparum* lysate (right panels) was probed with Pre-Immune (left lane) or anti-PfMyoF antibodies (right lane). **B** Co-immunoprecipitation from trophozoite-stage lysates identifies 25 enriched proteins; 15 were reproducible across three replicates and 9 across two replicates. **C** Gene Ontology (GO) analysis of the Co-IP hits shows enrichment in intracellular vesicles, secretory pathway and rhoptry-associated proteins. **D** A map of proteins enriched in the anti-PfMyoF Co-IP. Hits were defined as a 5-fold enrichment in iBAQ scores of anti-PfMyoF relative to the Pre-Immune control. Hits are arranged based on their predicted functions within the parasite. Solid border lines are for proteins identified in all 3 repeats, and dotted lines for proteins only found in one or two of the repeats.

To investigate PfMyoF expression, localisation and to identify potential interaction partners, we generated a polyclonal antibody against a 20 kDa fragment of the PfMyoF WD40 domain. This antibody, α-PfMyoF, was validated by immunoblotting against recombinant 6His-tagged WD40 antigen expressed in Sf9 cells and by detection of full-length endogenous PfMyoF in *P. falciparum* blood-stage lysates (Fig. 5A). Lower molecular weight bands likely reflect PfMyoF degradation products arising during sample processing.

We next performed co-immunoprecipitations (Co-IP) from late trophozoite lysates using α-PfMyoF and pre-immune serum as a control. PfMyoF was consistently precipitated in three independent experiments, confirming antibody specificity. Proteins enriched ≥5-fold, using intensity-based absolute quantification (iBAQ) scores as a measure of abundance relative to controls, were considered candidate interactors. This yielded 25 proteins, 15 of which were reproducibly detected across all three replicates and 9 proteins in at least two of the three replicates (Fig. 5B). Gene Ontology analysis revealed enrichment of proteins involved in membrane trafficking and protein secretion, including components of the ER, the ER-Golgi intermediate compartment, the Golgi complex, intracellular vesicles and rhoptries (Fig. 5C).

A map of all the proteins found in the PfMyoF Co-IP is shown in Fig. 5D. The strongest hit, PfRab18, is involved in early secretory trafficking and associates with vesicles at the ER, Golgi, and the parasitophorous vacuole membrane^40^. Two dynamin superfamily proteins, DrpC, a mitochondrial fission factor, and SEY1, an ER tubule-forming GTPase, were also identified. Furthermore, the cytoplasmic AAA+ ATPase CDC48 and its paralog PF3D7_0619400 at the apicoplast membrane were both enriched, suggesting a potential role for PfMyoF in ER-associated degradation (ERAD) and apicoplast-specific trafficking.

### PfMyoF localises to a perinuclear membrane compartment

**Fig. 6:**
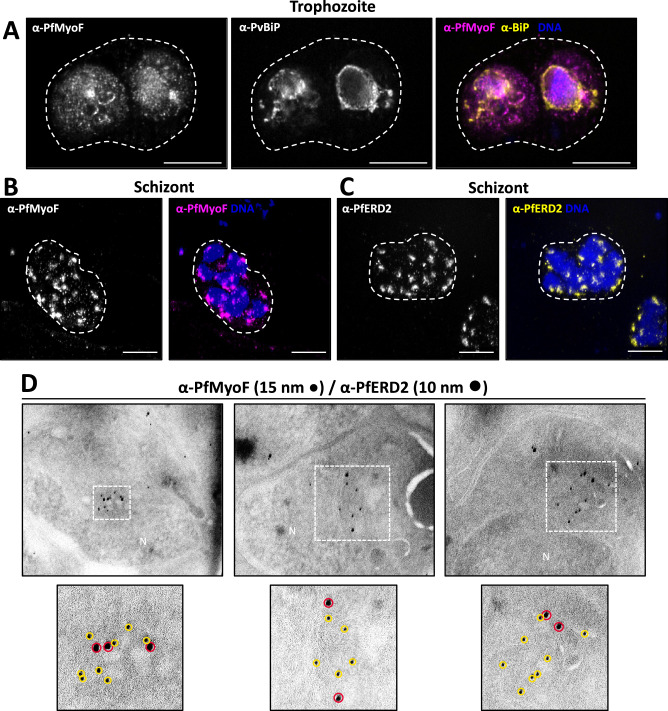
PfMyoF localises to the perinuclear region around the Golgi apparatus in *P. falciparum* parasites. **A** U-ExM of trophozoite stage parasites stained with anti-PfMyoF (magenta), the ER marker anti-PvBiP (yellow) and Hoechst (blue) shows enrichment of PfMyoF near the nucleus, partially overlapping with BiP (ER marker). **B** U-ExM of schizont stage parasites stained with anti-PfMyoF (magenta) and Hoechst (blue). PfMyoF remains concentrated in the perinuclear region. **C** U-ExM of schizont stage parasites stained with anti-PfERD2 (yellow) and Hoechst (blue) shows that PfMyoF localisation closely resembles that of the cis-Golgi marker PfERD2. All scale bars represent 10 µm. **D** Immunogold labelling of schizont stage parasites with anti-PfMyoF detected with Protein A conjugated 15 nm gold and anti-PfERD2 detected with Protein A conjugated 10 nm gold beads confirms co-localisation of PFMyoF and ERD2 on Golgi-proximal membranous structures. Red and yellow circles highlight 15 nm and 10 nm gold particles, respectively.

To further investigate the potential role of PfMyoF in intracellular transport, we examined the endogenous localisation of this myosin during *P. falciparum* blood-stage development using the α-PfMyoF antibody. Given the small size of *P. falciparum* parasites, ultrastructural expansion Microscopy (U-ExM) was performed to enhance resolution^41^. In trophozoite stage parasites, the antibody to endogenous PfMyoF revealed a predominantly perinuclear staining, enriched in a discrete region that partially colocalises with the endoplasmic reticulum marker BiP (Fig. 6A). In addition, a weak speckle-like PfMyoF signal was observed throughout the parasite cytoplasm. A similar staining pattern was also present in schizont-stage parasites, with one PfMyoF-enriched perinuclear dot per developing daughter merozoites in a schizont and a speckle-like distribution in the cytoplasm (Fig. 6B). Among the limited set of *P. falciparum*-specific antibodies available, the PfMyoF perinuclear staining pattern most closely resembled that of the cis-Golgi marker ERD2^42^ (Fig. 6C). Staining with pre-immune serum yielded no specific localisation within parasites, with only low-level background signal at the parasite periphery and surrounding red blood cells (Fig. S6).

We next examined PfMyoF localisations at ultrastructural resolution using immunogold electron microscopy. Schizont-stage parasites were embedded in gelatin, snap-frozen, and cut into ultrathin sections. These were sequentially labelled with α-PfMyoF detected with Protein A-15 nm gold conjugates, followed by α-ERD2 detected with Protein A-10 nm gold conjugates. In many parasites, PfMyoF (15 nm gold) and ERD2 (10 nm gold) were observed in close proximity on membranous structures adjacent to the nucleus^42^ (Fig. 6D). As ERD2 is a marker of the parasite Golgi complex, these results suggest that PfMyoF is present in the perinuclear region in close vicinity to the Golgi apparatus in *P. falciparum*. The additional cytoplasmic PfMyoF signal may correspond to Golgi-derived or Golgi-destined vesicles, although this remains to be confirmed.

In summary, these data demonstrate that PfMyoF localises predominantly to a perinuclear membrane compartment in trophozoite- and schizont-stage parasites, with partial overlap with ER and the cis-Golgi marker ERD2. The additional diffuse cytoplasmic PfMyoF signal may correspond to Golgi-derived or Golgi-destined vesicles, consistent with a role for PfMyoF in membrane trafficking pathways during the asexual blood stages of *P. falciparum*.

### Generation and phenotypic characterisation of PfMyoF conditional knockdown parasites

**Fig. 7:**
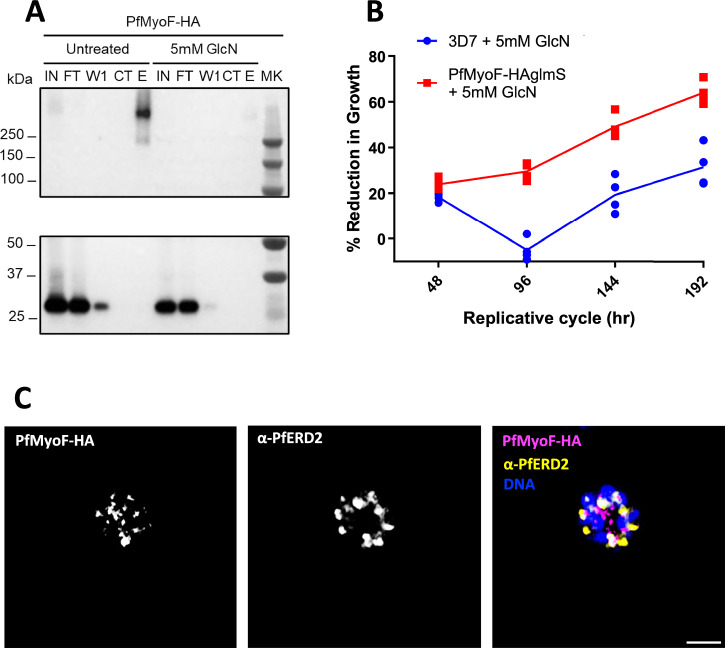
Conditional knockdown of PfMyoF impairs parasite growth. **A** HA-Trap affinity enrichment of PfMyoF-HA from PfMyoF-HAglmS parasites grown in the absence or presence of 5 mM glucosamine (GlcN). Blot shows enrichment of PfMyoF-HA from untreated parasites but is depleted following GlcN treatment. Anti-HA immunoblot (upper panel); anti-LDH antibodies were used as a loading control (lower panel). MK marker, IN input, FT flow-through, W1 wash 1, CT control bead eluate, E HA bead eluate. **B** The percentage reduction in growth across four replication cycles was compared between 3D7-wildtype and PfMyoF-HAglmS lines in the presence of 5 mM GlcN. To control for baseline variability, the untreated parasites for each line were used as its own internal control. Data are mean ± SEM for three replicates shown. *n* = 4. **C** PfMyoF-HA*glmS* parasites were stained with anti-HA (magenta), anti-PfERD2 (yellow) and Hoechst (blue). Scale bar: 2 µm.

To further analyse the function of PfMyoF during the asexual blood stage of the *P. falciparum* life cycle and to perform double labelling experiments of PfMyoF and the Golgi complex marker ERD2, we used CRISPR–Cas9-mediated genome editing to introduce a C-terminal 3×HA tag followed by the glmS ribozyme at the endogenous locus of PfMyoF in 3D7 parasites (Fig. S7A). The correct integration was confirmed by PCR using primers specific to the re-codonised region and the glmS sequence (Fig. S7B). PCR products of the expected sizes were detected only in PfMyoF-HAglmS parasites and not in wild-type 3D7 controls, confirming successful integration (Fig. S7B). Long-term culturing followed by repeated genotyping demonstrated that the PfMyoF-HAglmS line was genetically stable, replicating comparably to the wild-type 3D7 over multiple weeks in culture, with no evidence of growth defect or reversion of the modified locus to the wild-type locus. We induced conditional knockdown (KD) in the transgenic PfMyoF-HAglmS parasites using glucosamine. PfMyoF could not be consistently detected in whole-cell lysates by standard immunoblotting with α-PfMyoF or α-HA, presumably due to the low abundance of the protein. Therefore, HA-Trap affinity enrichment was used to enhance the PfMyoF-HA signal and assess depletion following GlcN treatment. In untreated parasites, PfMyoF-HA was readily enriched, whereas treatment with 5 mM GlcN resulted in near-complete loss of the HA signal in an HA-trap pull-down (Fig. 7A). These results confirm efficient and inducible depletion of PfMyoF protein upon activation of the glmS ribozyme.

To assess the functional impact of PfMyoF depletion, the growth of the transgenic parasites was monitored over multiple cycles under continuous GlcN treatment. PfMyoF-HAglmS parasites showed a clear, GlcN concentration and time-dependent growth defect that became pronounced after 96 h (two replication cycles) and even more pronounced after 192 h (four replication cycles) with a 64% reduction in growth (Fig. 7B). Wild-type 3D7 parasites showed a smaller reduction around 30% in growth at 5 mM GlcN over the 4 replication cycles most likely reflecting GlcN toxicity, which is a known limitation of the glmS system in *Plasmodium* parasites (Figs. 7B and S8). In summary, these results show that the conditional knockdown of PfMyoF using the glmS ribozyme causes a significant, glucosamine-dependent growth defect during the asexual blood stage. This demonstrates that PfMyoF is required for efficient parasite replication and is functionally important for blood-stage viability.

We also used the transgenic PfMyoF-HA*glmS* parasite line to perform double labelling experiments using monoclonal antibodies to HA and polyclonal antibodies to PfERD2. Immunofluorescence staining with anti-HA revealed a punctate perinuclear distribution that closely matched the pattern observed for endogenous PfMyoF (Figs. 6B and 7C). Co-staining with anti-ERD2 demonstrated partial spatial overlap, further supporting the conclusion that PfMyoF is enriched near Golgi-associated membranes in the perinuclear region (Fig. 7C).

## Discussion

This study provides the first comprehensive characterisation of PfMyoF, a divergent class XXII myosin in *P. falciparum*, identifying structural as well as functional features. Based on AlphaFold modelling, we propose that PfMyoF is a plus-ended, processive motor with the potential to dimerise and to adopt a folded autoinhibited conformation. PfMyoF contains two unique tail domains: a Rab-like and a WD40 domain, which together with its interactome and subcellular localisation support a role in membrane trafficking.

Within the neck domain, we show that PfMyoF binds PfCaM, the canonical calmodulin homologue in *P. falciparum*. Given the function of Myo-CaM interactions in other eukaryotes, these interactions are likely to stabilise the lever arm and support motor function. Processive motility within RPE cells, in the absence of PfCaM co-expression, suggests PfMyoF can also utilise human calmodulin, which shares 89% homology with PfCaM.

Using AlphaFold modelling, sequence alignments, and a live-cell filopodia assay, we establish PfMyoF as a plus-end-directed motor. This directionality has important implications for PfMyoF function in the parasite. Its plus-end directed, processive motility, together with its essential role in nutrient acquisition, suggests that PfMyoF may mediate the transport of vesicles through the parasite cytoplasm, likely towards the plasma membrane or organelles such as the Golgi complex^43^. However, the organisation and polarity of actin filaments in *Plasmodium* remain poorly defined^44^ and determining orientation will be essential to clarify the functional significance of PfMyoF directionality, especially considering the presence of two predicted minus-end-directed myosins, PfMyoJ and PfMyoK, in *Apicomplexa*.

Our analysis also supports the capacity of PfMyoF to dimerise and undergo autoinhibition. Although biochemical confirmation of dimer formation and autoinhibition is still required, the combined AlphaFold-3-based modelling and cell-based assays suggest that PfMyoF activity may be regulated through backfolding through interactions between the N-terminal SH3-like domain and the WD40 domain. These regulatory mechanisms are similar to those seen in mammalian myosins, including myosins of class V and VI^19,20^.

PfMyoF contains two unique tail domains not found in human or other *Plasmodium* myosins. The Rab-like domain appears to be a non-catalytic, pseudo-GTPase that may function in vesicle docking or Rab-effector recruitment. The PfMyoF WD40 β-propeller domain localises to the centrosome when expressed in mammalian cells, consistent with centrosomal targeting of TgMyoF in *T. gondii*^13^.

Co-immunoprecipitation experiments identified several interactors involved in membrane trafficking, dynamin-like proteins and actin regulators. These results further support a conserved role for PfMyoF in vesicle-associated processes, consistent with findings from *T. gondii*, where TgMyoF functions in organelle positioning and trafficking^31^. PfRab18, the most enriched interactor, is associated with vesicles destined for the parasitophorous vacuole membrane and exhibits perinuclear clustering with additional diffuse foci, a localisation pattern that is remarkably similar to endogenous PfMyoF^45^.

The growth defects of around 64% observed upon PfMyoF knockdown provide strong evidence that PfMyoF performs a function that is essential for sustained parasite replication. The growth defect of around 30% observed in the replication of wild-type parasites is consistent with GlcN toxicity described in previous studies in which the glmS ribozyme has been used to conditionally deplete *P. falciparum* proteins^46,47^. Interestingly, growth impairment in PfMyoF-HAglmS parasites was delayed and only became evident after multiple replication cycles. This may indicate that PfMyoF is a long-lived protein and that effective depletion may require several cycles or alternatively, or that loss of PfMyoF does not result in an immediate defect but instead disrupts processes whose impact accumulates over time. This pattern is consistent with defects in intracellular trafficking, organelle positioning or inheritance, which may accumulate over multiple replicative cycles rather than causing acute failures in bulk parasite growth. This conclusion is further supported by our co-immunoprecipitation results and previous interactome data as well as genome-wide association studies (GWAS) linking PfMyoF to endocytosis pathways involved in antimalarial drug resistance^12,48,49^.

In summary, our findings establish that PfMyoF possesses a specialised protein architecture allowing for processive motility, supporting a central role in intracellular cargo transport in *P. falciparum*. Its unique Rab-like and WD40 domains, plus-end directionality, predicted dimerisation, and potential autoinhibition collectively point to a central role in vesicular trafficking. This study characterises the key structural and functional features of PfMyoF, laying the groundwork for future investigations into cargo recognition, regulation by autoinhibition, and the roles of its unique Rab-like and WD40 tail domains. Our work provides a critical framework for future studies aimed at dissecting PfMyoF’s function and its potential as a therapeutic antimalarial drug target. The expression of class XXII myosins appears to be restricted to apicomplexan parasites, which are, to our knowledge, absent from higher eukaryotes, including humans, highlighting PfMyoF as an attractive candidate for selective therapeutic intervention. The divergence of PfMyoF from host myosins is not limited to its phylogenetic classification but extends to the presence of parasite-specific cargo-binding domains, which may present multiple opportunities for pharmacological targeting.

### Inclusions and ethics statement

This study did not involve human participants or live vertebrate animals. Ethical approval for the use of human blood was obtained from National Health Service (NHS) Cambridge South Research Ethics Committee (20/EE/0100), and the University of Cambridge Human Biology Research ethics committee (HBREC.2019.40). Formal written consent was obtained at the point of collection and is held by NHS Blood and Transplant (NHSBT).The research was conducted in accordance with relevant institutional guidelines and regulations.

## Methods

### Sequence alignment

Sequences for PfMyoF (PF3D7_1329100), PfMyoJ (PF3D7_1229800) and PfMyoK (PF3D7_1140500) and other *P. falciparum* proteins were curated from PlasmoDB (https://plasmodb.org/plasmo/app). Multiple Sequence Alignments were performed using MUSCLE software (EMBL)^50^ and Pairwise Alignments performed using EMBOSS Stretcher (EMBL)^51^.

### AlphaFold-3 structural prediction

AlphaFold Prediction and Structural Homology Protein structures were predicted using AlphaFold-3 software. Structures are coloured by the strength of prediction—with dark blue areas equalling high confidence and yellow/orange areas equalling low confidence. The Predicted Aligned Error (PAE) matrices shown can also be used to assess AlphaFold’s confidence in its prediction. The PAE plot represents a residue-by-residue error estimate matrix where both axes correspond to residue positions in the protein sequence. Darker green region in the matrix indicates high confidence interaction and lighter/white regions indicate low confidence. Squares of green indicate the presence of a protein domain. In addition, PAE plots are useful for examining predicted protein-protein interactions. To identify structural homologues, protein structures were saved as PDB files and uploaded to the DALI server for testing against both the PDB server and AlphaFold 2 databases for *H. sapiens* and *P. falciparum* and sorted by *Z*-score (a measure of similarity and significance).

For AlphaFold prediction of neck-light chain interactions, ten calmodulin and calmodulin-like proteins were identified using the PlasmoDB database: PfCaM (PF3D7_1434200); ELC (PF3D7_1017500); Putative CaM (PF3D7_1418300); Putative CaM (PF3D7_1030800); PF3D7_0714400 (PF3D7_0714400); PLC-3 (PF3D7_0414200); EF-hand calcium-binding domain-containing protein (PF3D7_1444200); EF hand domain-containing protein (PF3D7_0728500); calcium-binding protein (PF3D7_0605400); PLC-5 (PF3D7_0627200).

When predicted using AlphaFold, pTM scores ranged from 0.46 to 0.81, with only two proteins (PF3D7_0728500 and PF3D7_0714400) below the required >0.5 pTM score suggestive of a true-to-life model. Despite this, all putative light chains produced high-confidence EF-hand domains responsible for IQ binding. PfCaM achieved a pTM score of 0.58.

### Mammalian cell culture

Human Retinal Pigment Epithelial (hTERT RPE-1, ATCC, CRL-4000) cells were maintained in Dulbecco’s Modified Eagle’s medium (D6546, Sigma Aldrich)—HAM’s F-12 Nutrient Mix (N4888, Sigma Aldrich) supplemented with 2 mM L-glutamine, 100 U/mL penicillin 100 µg/mL streptomycin and 10% fetal bovine serum at 37 °C with 5% CO_2_. Cells were washed with phosphate-buffered saline (PBS, Sigma), incubated with 0.05% trypsin-EDTA for a few minutes (Sigma) and transferred to a 15 mL tube in DMEM Buffer. Cells were passaged by dilution into fresh DMEM media in a new flask.

RPE cells were seeded onto glass coverslips in six-well plates (typically 80,000 cells per coverslip), so that cells reached near confluency the following day. After settling and adhering to the coverslip, cells were transfected using FuGene® 6 transfection reagent as per manufacturer’s protocol. DNA (2 µg) and Fugene (6 µl) were made up to a total of 100 µl in Opti-MEM, mixed briefly, and incubated for 15 min at room temperature. The DNA-FuGene mix was then added dropwise to the cells.

### *P. falciparum* parasite culturing

*P. falciparum* 3D7 cell line was obtained from BEI Resources (MRA-102) and cultured in washed erythrocytes in modified RPMI media (R0883, Sigma-Aldrich) supplemented with 0.5% Albumax II, 0.2 mM hypoxanthine, 25 mM HEPES, sodium bicarbonate, and 12.5 µg/ml gentamycin). Cells were grown in a 37 °C incubator with 5% CO_2_. During routine culturing, parasitaemia was maintained below 5%. Parasitaemia was assessed every 2 days by Giemsa-smear. Parasites were synchronised by sorbitol lysis. Infected erythrocytes were pelleted by centrifugation and resuspended in 5% sorbitol. After 5 minutes of incubation at 37 °C, cells were washed in PBS and returned to cultures. For microscopy, parasites were fixed in 4% paraformaldehyde with or without 0.75% glutaraldehyde. To prepare parasite lysates for immunoblotting, parasites were pelleted and lysed with 0.1% saponin in ice-cold PBS supplemented with 1X Complete protease inhibitor (Sigma-Aldrich).

### Generation of PfMyoF-HAglmS transgenic *P. falciparum* lines

To generate conditional knock-down parasites, re-codonised 3’ region of PfMyoF, a C-terminal 3xHA epitope tag and the *glmS* ribozyme sequence were PCR amplified (see Table 1) and cloned into the pUC19 vector using *EcoR*I and *Hind*III restriction sites, generating a fusion of the desired gene of interest under the control of the native *PfMyoF* promoter. The gRNAs targeting the PfMyoF locus were cloned into the pDC-Cas9 plasmid. Synchronised *P*. *falciparum* wild-type 3D7 ring-stage parasites at approximately 5% parasitaemia were transfected with a total of 90 µg plasmid DNAs (30 µg gRNA plasmid and 60 µg repair template) by electroporation (310 V, 950 μF). Transfected parasites were selected using DMS1 (1.5 µM). Correct genomic integration was confirmed by PCR using primers specific to the re-codonised region and the *glmS* sequence (see Table 1). Expression of the PfMyoF-HA fusion protein in the transgenic line was assessed by immunoblotting following HA-Trap affinity enrichment of parasite lysates and detection with anti-HA tag antibodies.

**Table 1:** Primers used for generating the PfMyoF-HAglmS construct

Name	Sequence
3’MyoF G1 F	ATTGTTTTATTTAGGCACCAGTAA
3’MyoF G1 R	AAACTTACTGGTGCCTAAATAAAA
3’MyoF G2 F	ATTGGTCATGTCAGGTTCAGAGGA
3’MyoF G2 R	AAACTCCTCTGAACCTGACATGAC
3’MyoF G3 F	ATTGTATATTATATTATATGTATG
3’MyoF G3 R	AAACCATACATATAATATAATATA
MyoF Flank F	GGTTTACAACCGGTTTATGAAATAACGC
MyoF Flank R	CCACCTAGGCATATAAATCAATACCAC
MyoF Genotype WT F	GTTTTCTGGCGCAGGTCTTTTG
MyoF Genotype WT R	CCGCCACCAAACCTGGAATAAC
MyoF HR1 F	cgttgtaaaacgacggccagtgaattcATGAACAGCAATACCACCCTGAATAT
MyoF HR1 R	AAACCGAGAGTAGCAGTTCCTTATGGGAAGTAAGGCATGATTTATTCG
MyoF HR2 F	CAAAGAGAAATCACATGATCTGTGTATACATTTGAATATATTCCTATGTATTTATTTTTAAGTG
MyoF HR2 R	gctatgaccatgattacgccaagcttTACGTCAAAGTGTTATATATAAACAACAACAAAAATGTTG
MyoF NOTAG HR2 F	GGCGCAGAAGTTTCGTGTGAGTGTATACATTTGAATATATTCCTATGTATTTATTTTTAAGTG
MyoF NOTAG Twist R	TCACACGAAACTTCTGCGCCAAA
MyoF Twist F	AGGAACTGCTACTCTCGGTTTGG

### Flow cytometry and growth assays

Parasite growth was quantified by measuring the parasitaemia of PfMyoF-HA*glmS* parasites relative to wild-type 3D7 parasite controls. Synchronised ring-stage 3D7 and PfMyoF-HA*glmS* parasites were seeded at low parasitaemia (∼0.3%) and grown at 4% haematocrit in complete parasite media supplemented with varying concentrations (1 mM, 2.5 mM, and 5 mM) of glucosamine. Experiments were set up in triplicate and repeated four times. To allow monitoring of parasitaemia up to the fourth replication cycle and prevent cultures from overgrowing to high parasitaemia, cultures were diluted 1:40 after replication cycle 2 (96 h). To determine the parasitaemia, 10 µL of infected red blood cells (iRBCs) were stained with SYBR Green I (Invitrogen) diluted in PBS. At least three wells containing uninfected RBCs at the same haematocrit were used as negative fluorescence controls^52^. The SYBR Green-stained cells were incubated at 37 °C for 30–45 min in the dark, washed once, resuspended in PBS and analysed using a 488 nm blue laser on an Attune Nxt Flow Cytometer (Thermo Fisher Scientific) equipped with a 96-well plate autosampler (CytKick). A total of 100,000 events per well were recorded at a flow rate of 100 µL/min from an acquisition volume of 100 µL. Attune Nxt Software was used to plot forward scatter (FSC-A) versus side scatter (SSC-A) to gate for uninfected RBCs, FSC-H vs. FSC-A to exclude doublets, and FSC-H vs. BL1 (SYBR Green I) to distinguish infected from uninfected RBCs and to calculate the parasitaemia. Parasitaemia values of PfMyoF-HA*glmS* parasites were compared with untreated controls and 3D7 wild-type parasites using two-way ANOVA. Graphs were generated in GraphPad Prism (9.1.2 (226). Percentage growth reduction was calculated by normalising parasitaemia values from glucosamine-treated cultures to matched untreated controls at the same timepoint, using arithmetic mean values. This is expressed mathematically as follows:

$${{\mathrm{Percent}}}\,{{\mathrm{reduction}}}=\left(1-\frac{{{\mathrm{Parasitaemia}}}\,{GlcN}-{treated}}{{{\mathrm{Parasitaemia}}}\,{Untreated}}\right)\times 100.$$

### *E. coli* protein expression

For expression in *E. coli*, GST-tagged PfMyoF WD40 fragments and GST-GFP nanobodies were cloned into a bacterial expression vector containing a tag for subsequent purification (either a 6-His tag in pRSETA or a GST tag pGex-4T). Expression vectors were transformed into BL21 cells, a single colony was picked and grown overnight in 5 mL 2xTY plus ampicillin at 37 °C. Frozen stocks of the transformed bacteria culture were made by the addition of 20% glycerol (1:1 ratio) and stored at −80 °C. To express protein, a smear of frozen stock was inoculated in 50 mL 2xTY media plus ampicillin and grown overnight at 37 °C at 200 RPM. The following day, the 50 mL culture was added to 1 L 2xTY plus ampicillin and grown at 37 °C for 2–4 h until the OD_600_ reached 0.6–0.8. OD_600_ values were measured on a Jenway 7305 UV/Visible Spectrophotometer. Once the required OD_600_ value was reached, the temperature was lowered to 22 °C and the culture allowed to cool for 30 min. Cultures were then induced using 0.5 mM IPTG and grown overnight at 22 °C at 200 RPM.

### Sf9 protein expression

Coding sequences of the genes of interest were optimised for expression in *Spodoptera frugiperda* (Sf9) cells, ordered as a g-block and cloned into pFastBac1 vectors. Constructs were then cloned into bacmids, large plasmids containing genes for baculovirus expression, using BAC10 cells. ExpiSf9 cells (Thermo Fisher Scientific, A35243) were maintained at 27 °C, 125 rpm in Sf9 ExpiSf™ CD Medium (A3767801, Gibco™) at a density between 1 ×106 and 1 ×107 cells/ml. Proteins were expressed from direct transfection of bacmid using ExpiFectamine or through viral infection. Cells were transfected using ExpiFectamine SF transfection reagent (A38915, Gibco) according to the ExpiSf™ Expression System user guide (MAN0017532). Transfected cells were grown for 48–96 h. Cells were pelleted at 310 × *g* for 5 min. The pelleted cells were stored or used immediately for protein purification.

### Protein purification

Cells were pelleted and supernatant discarded. The pellet was resuspended in lysis buffer (20 mM Tris pH 8.0, 500 mM NaCl, 10 mM MgCl_2_, 1 mM EGTA, 1 mM TCEP, protease inhibitors (Roche cOmplete Mini, EDTA-free), 0.1% Triton X-100, 5 mM ATP), sonicated and centrifuged at 40,000 RPM at 4 °C for 35 min in a Type 45 Ti rotor within an Optima XPN 90 K ultracentrifuge. The resultant supernatant was carefully removed and filtered through a 0.22 µm filter. Glutathione Sepharose™ 4B (Cytiva) beads were washed three times in wash buffer, added to the cleared lysate and rocked end-over-end for 4 h at 4 °C. In the case of GFP-tagged proteins, a GST-GFP nanobody was also added to the cleared lysate. Beads were then pelleted by centrifugation at 2600 RPM for 5 min at 4 °C and washed three times in wash buffer (20 mM Tris, pH 8.0, 300 mM NaCl, 5 mM MgCl2, 1 mM EGTA) to remove nonspecific interactions. GST-tagged protein was eluted using the elution buffer (20 mM Tris pH 8.0, 300 mM NaCl, 5 mM MgCl2, 1 mM EGTA, 50 mM Reduced L-Glutathione) by incubating end-over-end for 20 min to 2 h. The GST-GFP nanobody was removed from the GST-tagged protein using PreScission Protease, and the released PfMyoF was isolated by incubation with Glutathione Sepharose™ 4B beads. Protein concentration was measured using the Precision Red Advanced Protein Assay (ADV02, Cytoskeleton) and proteins of interest identified by SDS-PAGE analysis.

### SDS-PAGE

Denatured protein samples were resolved using Sodium Dodecyl Sulfate–PolyAcrylamide gel Electrophoresis (SDS-PAGE). Proteins were denatured by boiling for 5–10 min at 95 °C in 5X sample buffer and loaded onto NuPAGE 4–12% Bis-Tris protein gels (NP0332) in NuPAGE 1X MOPS running buffer (NP0001) alongside Precision Plus Protein™ Standards ladder (Bio-Rad). Gels were run at a constant voltage of 200 V for 45 min at room temperature by an electrophoresis PowerPac™ (Invitrogen). Separated protein samples were visualised using InstantBlue™ Coomassie stain or processed for Western Blotting.

### Western blotting

Specific proteins of interest were identified by Western Blot. Proteins first separated by SDS-PAGE were transferred onto a nitrocellulose membrane or a methanol-activated polyvinylidene difluoride (PVDF) membrane (Immobilon-P Millipore). Transfer took place in ice-cold 1X transfer buffer at 350 mA using an electrophoresis PowerPac™ (Invitrogen). Post-transfer, the membranes were blocked in 5% milk solution for 1 h to prevent non-specific binding. The membranes were then incubated with primary antibodies diluted in PBST overnight before washing in PBST for 3 ×10 min. After incubating with secondary antibodies for 1.5 h at room temperature and washing in PBST for 3 ×10 min, membranes were incubated with enhanced chemiluminescent (ECL) detection reagent (GE Healthcare) for 1 min and exposed to-ray film (Fujifi).

### Generation of polyclonal antibodies

GST-tagged PfMyoF WD40 fragments expressed and purified from *E. coli*, underwent size exclusion chromatography and were immunised by Eurogentec using their ‘classic’ antibody production programme alongside Freund’s complete adjuvant. The immunisation programme lasted 87 days in total.

### Co-immunoprecipitation and protein pulldown

A total of 100 ml of *P. falciparum* parasite cell cultures were grown to 5% parasitaemia and spun down for 5 min. Red blood cells were lysed by resuspension in 0.1% saponin made up in ice-cold PBS and complete protease inhibitors. After 5 min, the remaining parasites were pelleted by spinning for 3 min at 3000 × *g* at 4 °C. The pellet was resuspended in ice-cold PBS + protease inhibitor and underwent repeated washes until all traces of heme/blood were removed. The parasite pellet was then resuspended in the lysis buffer (50 mM Tris pH 7.4, 150 mM NaCl, 5 mM MgCl2, 1% NP-40, 5 mM ATP, complete protease inhibitor (Roche cOmplete Mini, EDTA-free)) and incubated for 30 min on ice with occasional gentle pipetting. The cell lysate was passed through a 21 G needle 3 times to shear DNA before centrifugation at 14,000 RCF to remove cellular debris. The resulting supernatant was incubated with Protein A end-over-end at 4 °C for 45 min to remove protein non-specifically bound to the Protein A beads. Protein A beads were then added alongside 5 µg of antibody and incubated end-over-end for 90 min at 4 °C. Beads were then pelleted and washed three times in the lysis buffer. All supernatant was removed with a U-100 insulin syringe, and the remaining beads were then boiled in sample buffer.

### Mass spectrometry

All co-immunoprecipitation samples were analysed using suspension trapping (S-Trap) at the Cambridge Institute for Medical Research (CIMR) proteomics core facility. Samples were lysed in 5% sodium dodecyl sulfate (SDS) in 50 mM triethylammonium bicarbonate (TEAB), reduced in 10 mM dithiothreitol (DTT) at 56 °C for 30 min and akylated with 20 mM iodoacetamide (IAA) at room temperature for 30 min. Proteins were digested overnight at 37 °C with Trypsin/Lys-C protease mix (Promega) in 50 mM TEAB at an enzyme to protein ratio of 1:20 (w/w). Peptides were eluted off the S-Trap column by sequential washes of 40 μl 50 mM TEAB, 40 μl 0.2% formic acid (FA) in water and 40 μl 0.2% FA in 50% acetonitrile. Eluates were pooled, dried in a SpeedVac and resuspended in MS loading solvent (3% acetonitrile, 0.1% trifluoroacetic acid (TFA)). Peptides were analysed on a Q Exactive Plus mass spectrometer (Thermo Fisher Scientific) coupled to an Ultimate 3000 RSLCnano UPLC system (Thermo Fisher Scientific). Separation was performed on a 50 cm C18 PepMap EASY-Spray column using a gradient from 3% to 40% solvent B over 70 min (solvent A: 0.1% formic acid in water; Solvent B: 80% acetonitrile, 0.1% formic acid). Data were acquired in a Top10 data-dependent acquisition mode. Full MS spectra were collected over an *m*/*z* range of 400–1500 at 70,000 resolution (FWHM) and MSMS spectra were acquired at 17,500 resolution with a dynamic exclusion 30 s. Raw files were processed as a single batch using the MaxQuant (version 2.6.7.0). Spectra were searched against the reviewed Uniprot Plasmodium falciparum database (downloaded 04/2024).

The mass spectrometry proteomics data have been deposited to the ProteomeXchange Consortium via the PRIDE^53^ partner repository with the dataset identifier PXD077305.

### Light microscopy

RPE Cells were fixed in 4% formaldehyde for 20 min, permeabilised in 0.2% Triton-100 for 2 min, blocked in 4% BSA/PBS for 60 min, and labelled with primary and secondary antibodies alongside Phalloidin and Hoechst. Coverslips were placed cell-side down onto ProLong Gold Antifade Reagent. Images were acquired using Zeiss AxioImager Z2 and LSM880 Airyscan confocal microscopes.

### Filopodia direction assay

RPE cells were transfected with equal quantities of mCherry-tagged MYO6+ (a chimeric, plus-end-directed MYO6) and GFP-tagged myosin constructs under investigation for directionality. After 20 h, cells were fixed in 4% PFA and stained with anti-GFP, anti-RFP, Hoechst and Phalloidin-647.

Coverslips were blinded prior to imaging using a Zeiss AxioImager Z2. For each GFP-tagged construct, 300 MYO6+ positive filopodia were analysed (100 filopodia per coverslip, across three coverslips). GFP signal was scored by eye as localising to the tip, shaft or being absent of filopodia based on reference to the archetypes established for MYO5A, MYO1E and MYO6 (Fig. S4). Localisation percentages are presented in bar charts with standard error of the mean (SEM) shown.

### Ultrastructural expansion microscopy (U-ExM)

Ultrastructural expansion microscopy (U-ExM) and Iterative U-ExM (iU-ExM) typically result in a 5X and 20X enlargement of a microscopy sample, respectively. All U-ExM and iU-ExM experiments were performed as detailed in refs. ^24^^,^^29^, respectively. In short, for U-ExM, washed *P. falciparum-*infected erythrocytes were fixed in 4% formaldehyde for 30 min at room temperature. Fixed parasites were then allowed to settle onto poly-D-lysine-coated 12 mm coverslips for 1 h. Coverslips were incubated in 1 ml anchoring solution (1.4% Formaldehyde, 2% acrylamide, and Sodium acrylate 38% w/v in 1 X PBS) overnight at 37 °C in 12-well dishes sealed with parafilm. The following day, a humid gelation chamber was assembled and placed in a −20 freezer 20 min prior to gelation. Monomer solution (19% w/v Sodium acrylate, 10% w/v Acrylamide, 0.1% w/v Bis (N, N’-methylenebisacrylamide)), 10% TEMED and 10% APS solutions were thawed in an ice-cold metal Eppendorf holder. Monomer solution (90 µl) was activated by the addition of TEMED (5 µl) and APS (5 µl), quickly vortexed, and 35 µl pipetted onto the parafilm layer of the gelation chamber. The anchored sample was quickly blotted to remove excess anchoring solution and placed cell-face down onto the active monomer solution. Gelation tanks were left on ice for 5 min to allow complete absorption of the solution by the cells and then placed at 37 °C for 1 h, during which the gel is polymerised. For U-ExM, gels were boiled in 1.5 ml denaturation buffer (200 mM SDS, 200 mM NaCl, 50 mM Tris in pure water, pH 9.0) at 95 °C for 30 min. After denaturation, gels were hydrated in ultrapure water and expanded overnight at 4 °C. Water was completely replaced three times within the first hour of hydration to remove residual salt from the denaturation buffer. To immunolabel, 2 × 2 cm^2^ gel fragments were cut, placed in a 6-well dish and dehydrated by three 1X PBS washes and blocked in 3% BSA for 1 h at 37 °C. Primary antibodies were diluted into 3% BSA and incubated overnight in 37 °C shaking rocker at 90 RPM. Gels were washed three times for 10 min in 1X PBST, followed by the addition of secondary antibodies and Hoechst diluted in 3% BSA. Gels were incubated with secondary antibody for a minimum of 5 h, shaking at 37 °C. Gels were once again washed three times in PBST. For U-ExM, gels were hydrated overnight and ready for imaging.

Gels were imaged using Airyscan confocal microscopy on an LSM880 microscope. A small section of gel was cut and blotted extensively with Kimwipes. The gel was then gently pressed onto a poly-D-lysine-coated coverslip using a paintbrush. Once the correct plane of focus was found, pure water was added to the imaging dish to prevent gel dehydration during imaging.

### Antibodies

α-PfMyoF (1/300, Rabbit, generated in this study); α-GFP (1/200, Rabbit, A-11122, Invitrogen); α-GFP (1/100, Mouse, 9F9.F9, Abcam); α-RFP (1/1000, Rat, Clone No. 5F8, Proteintech); α-γTubulin (1/250, mouse, Clone No. GTU-88, Santa Cruz); α-Acetylated-αTubulin (1/250, Mouse, sc-23950, Santa Cruz); α-HA (1/100, Rat, Roche 50 mg/mL stock); α-HA (1/500, Mouse, Sigma); α-Centrin (1/250, Mouse, Clone No. 20H5, Sigma), α-PvERD2 (1/500, Rabbit, gifted from the Rayner Lab), α-PvBiP (1/250, Rat, gifted from the Rayner Lab), Alexa Fluor-conjugated secondary antibodies (1/1000, Donkey & Goat, Invitrogen); Phalloidin 647 (1/1000, A22287, Invitrogen); Hoechst (1/1000, 33342, Invitrogen); Atto 565 NHS Ester (1/300, 72464, Sigma).

### Immunoelectron microscopy

Parasites were tightly synchronised to a 4-h window by repeated rounds of sorbitol and Percoll synchronisation. The egress inhibitor, Compound 2, was used to arrest mature schizonts for 4 h prior to Percoll purification and fixation with 4% paraformaldehyde/0.075% glutaraldehyde in PBS for 30 min at 37 °C. The cell suspension was then pelleted (13,000 rpm for 5 min), the fixative was aspirated, and the cell pellet was re-suspended in warm 10% gelatin. The cells were then pelleted (13,000 rpm for 5 min), and the gelatin-enrobed pellet was set on ice, trimmed into 1 mm 3 blocks and infused with 2.3 M sucrose for 24 h at 4 °C. The blocks were subsequently mounted on cryostubs and snap-frozen in liquid nitrogen. Frozen ultrathin sections (70 nm) were cut using a Diatome diamond knife in a Leica FC7 cryochamber attachment (Leica, Milton Keynes, UK), collected from the knife-edge with 50:50 2% methyl cellulose: 2.3 M sucrose and mounted on formvar-carbon coated EM grids.

Immunolabelling was performed using the protein A-gold technique at room temperature^54^. Sections were incubated with 50 mM NH_4_Cl in PBS for 10 min to quench unreacted aldehydes and then 1% BSA/5% FCS in PBS for 10 min. Sections were incubated for 30 min with 5 ml of PfERD2 antibody 1:50 in PBS containing 5% FCS and 0.1% BSA. The sections were washed with PBS/0.1% BSA (6 ×3 min) and incubated for 30 min with PBS/0.1% BSA containing protein A conjugated to 10 nm colloidal gold. The sections were washed with PBS/0.1% BSA (2 ×5 min), PBS (4 ×5 min) and the complex was stabilised using 1% glutaraldehyde in PBS (5 min).

Sections were incubated with 50 mM NH_4_Cl in PBS for 10 min to quench unreacted aldehydes and then 1% BSA/5% FCS in PBS for 10 min and were then incubated for 30 min with 5 ml of PfMyoF antibody diluted 1:50 in PBS containing 5% FCS and 0.1% BSA. The sections were washed with PBS/0.1% BSA (6 ×3 min) and incubated for 30 min with PBS/0.1% BSA containing protein A conjugated to 15 nm colloidal gold. The sections were washed with PBS/0.1% BSA (2 ×5 min), PBS (4 ×5 min). Finally, the sections were rinsed with distilled water (5 ×3 min) and contrasted by embedding in 1.8% methyl cellulose/0.3% uranyl acetate. Sections were allowed to air dry prior to observation in an FEI Tecnai G2 BioTWIN transmission electron microscope (FEI, Eindhoven) at an operating voltage of 80 kV. Images were recorded with an Eagle 4 K CCD camera.

### Statistics and reproducibility

All experiments were performed with at least three independent biological replicates unless otherwise stated. Biochemical assays, including co-purification and pull-down experiments, were repeated independently with similar results. Co-immunoprecipitation experiments were performed in three independent biological replicates, and proteins reproducibly enriched relative to controls were considered candidate interactors. Imaging experiments are representative of multiple independent experiments with consistent localisation patterns. For the filopodia directionality assays, more than 300 MYO6+-positive filopodia were analysed per construct across three coverslips, and localisation frequencies are reported as mean ± s.e.m. These experiments were performed blinded. Parasite growth assays were performed in triplicate and repeated four times independently. Statistical comparisons of parasitaemia were performed using two-way ANOVA in GraphPad Prism v9.

### Reporting summary

Further information on research design is available in the Nature Portfolio Reporting Summary linked to this article.

## Supplementary information

Supplementary Information

Description of Additional Supplementary Files

Supplementary Data 1

Reporting Summary

## Data Availability

All data supporting the findings of this study are available within the paper and its Supplementary Information files. Uncropped and unedited images of blots/gels in Figs. 1H, 5A, 7A and Supplementary Fig. 7B are shown in Supplementary Figs. 9–12. The numerical source data for Figs. 4E, 5D and 7B is shown in Supplementary Data 1. The proteomics data are available via ProteomeXchange with identifier PXD077305.
