# Supplementary material for: Plasmodium falciparum PfMyoF contains a Rab-like tail domain and associates with perinuclear membrane trafficking proteins
Source: Commun Biol. 2026 May 15;9:1056. doi: 10.1038/s42003-026-10192-1 (PMC13448217; doi:10.1038/s42003-026-10192-1)
Supplement: Supplementary file 1 [file 42003_2026_10192_MOESM1_ESM.pdf]

Supplementary Figure 1. AlphaFold-3 predictions of *P. falciparum T. gondii* myosins.

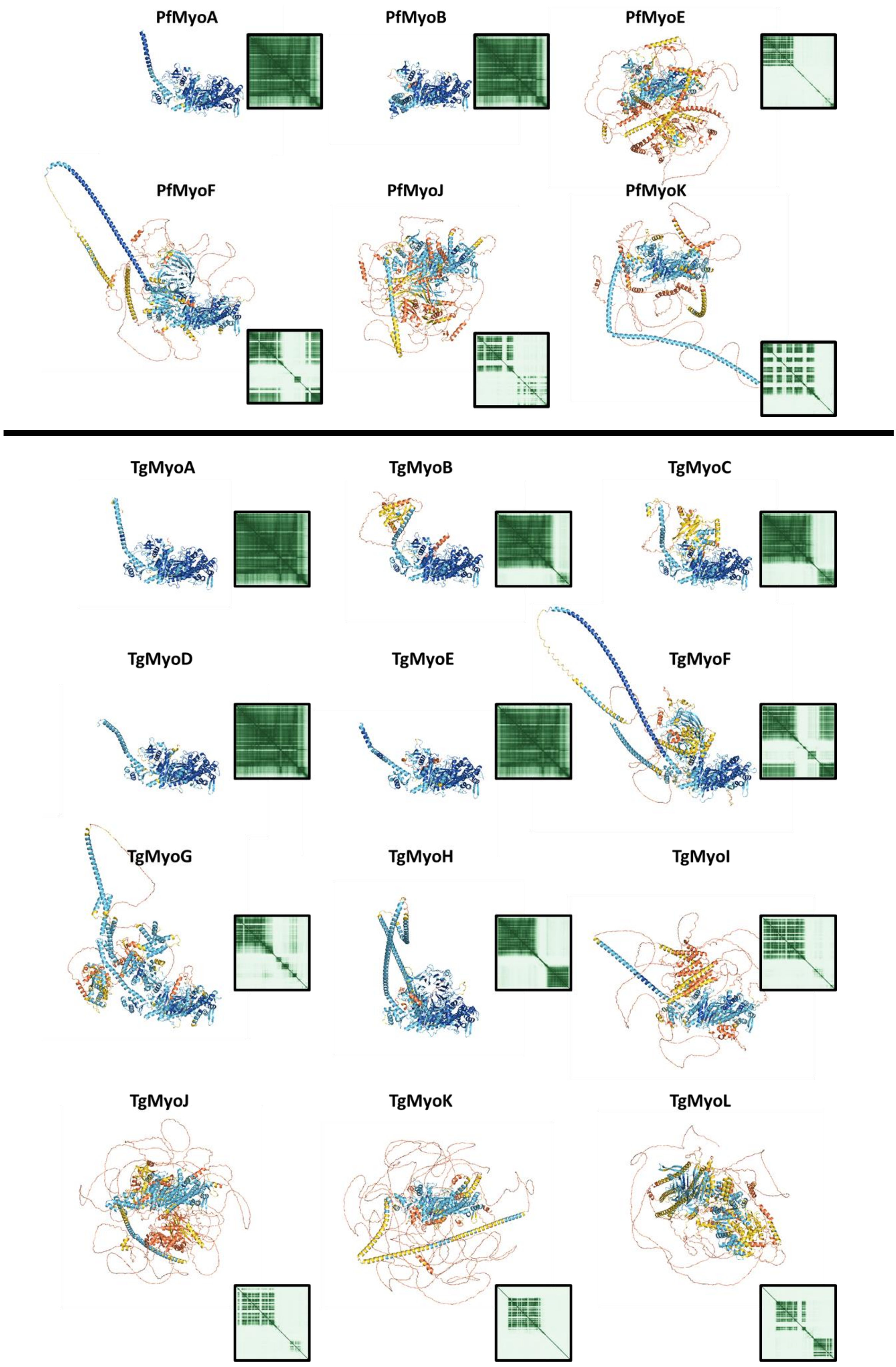

AlphaFold-3 predictions of the six *P. falciparum* myosins (top) and eleven *T. gondii* myosins (bottom). PAE graphs are shown to the right of each structure.

Supplementary Figure 2. The Annotated PfMyoF sequence

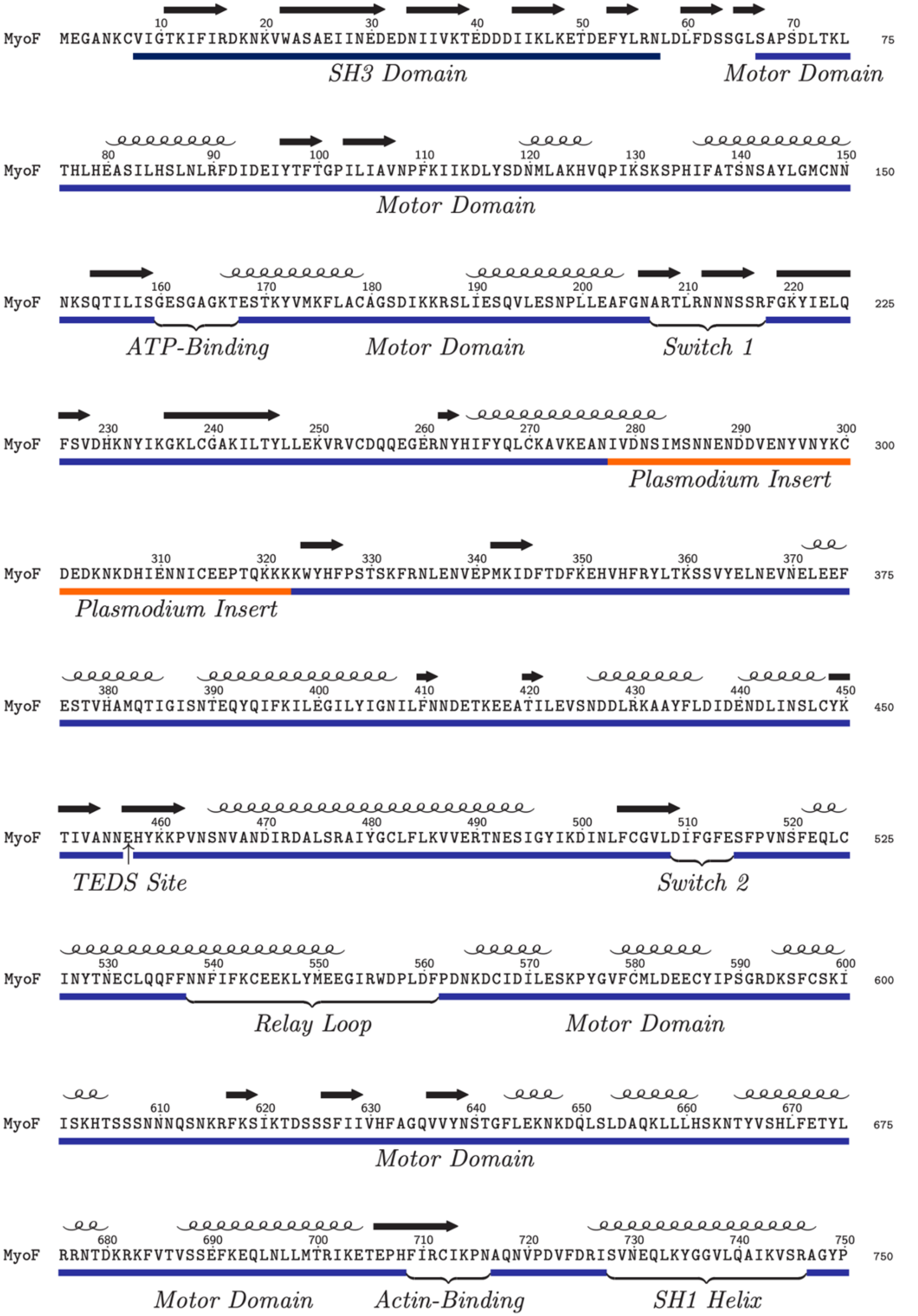

2A The Annotated PfMyoF Motor domain. Alpha helices are labelled as such and β sheets as black arrows. Orange sequences highlight large asparagine-rich Plasmodium specific insertions.

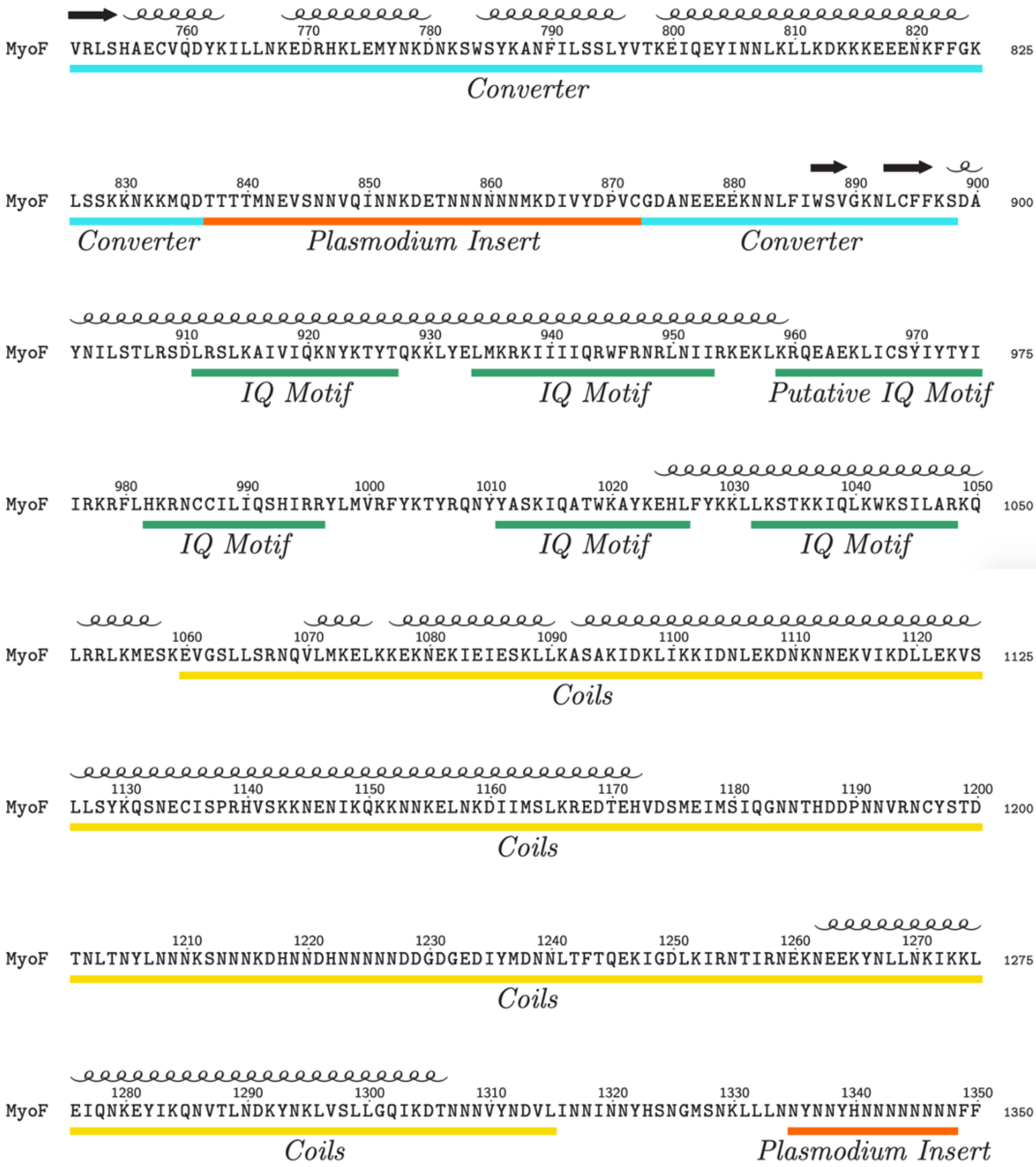

2B. The Annotated PfMyoF Converter, Neck and Coil domains. Alpha helices are labelled as such and β sheets as black arrows. Orange sequences highlight large asparagine-rich Plasmodium specific insertions.

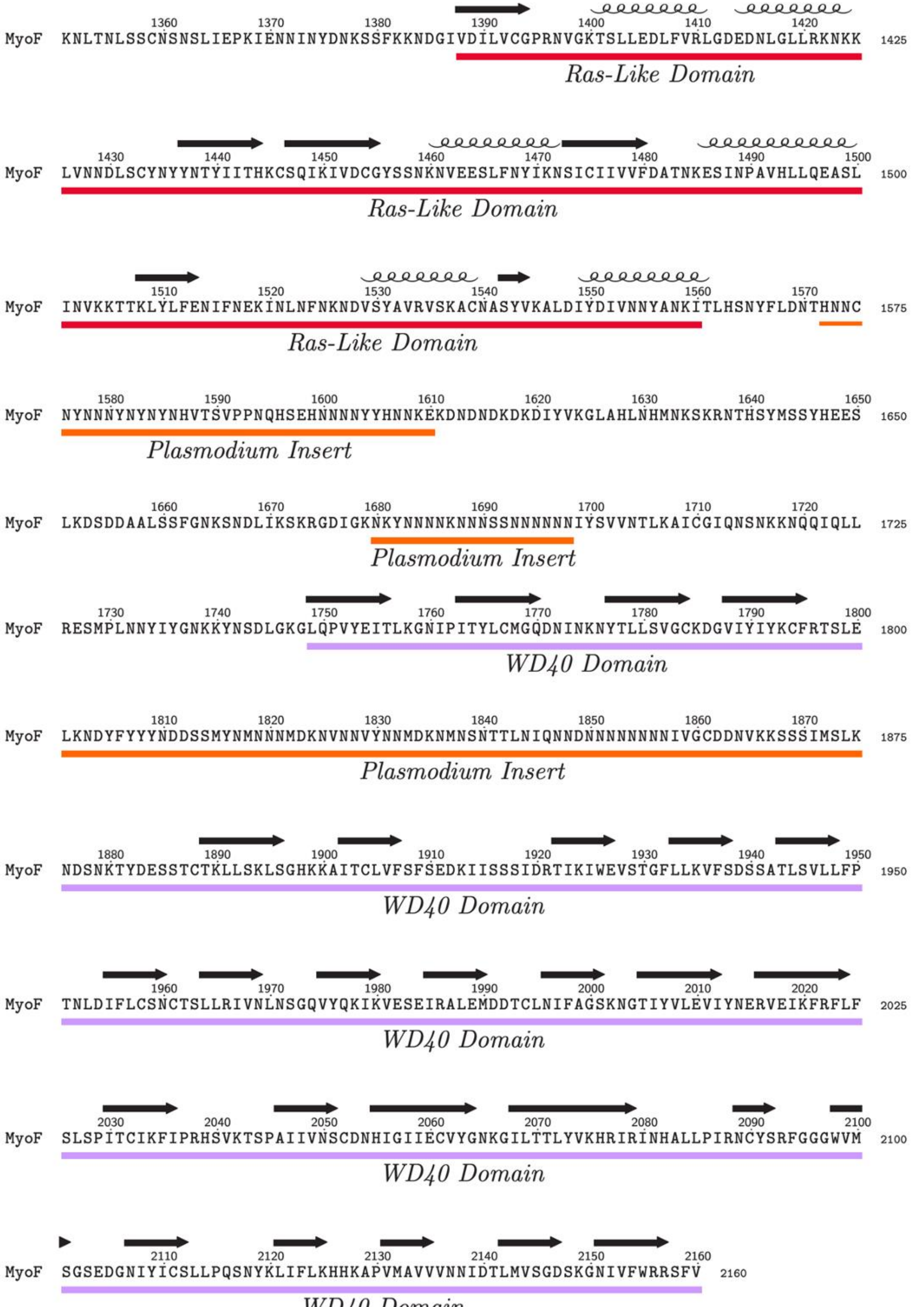

2C . The Annotated PfMyoF Tail domain. α-helices are labelled as such and β-sheets as black arrows. Orange sequences highlight large asparagine-rich Plasmodium specific insertions.

Supplementary Figure 3. AlphaFold-3 predictions of the PfMyoF Rab-Like domain.

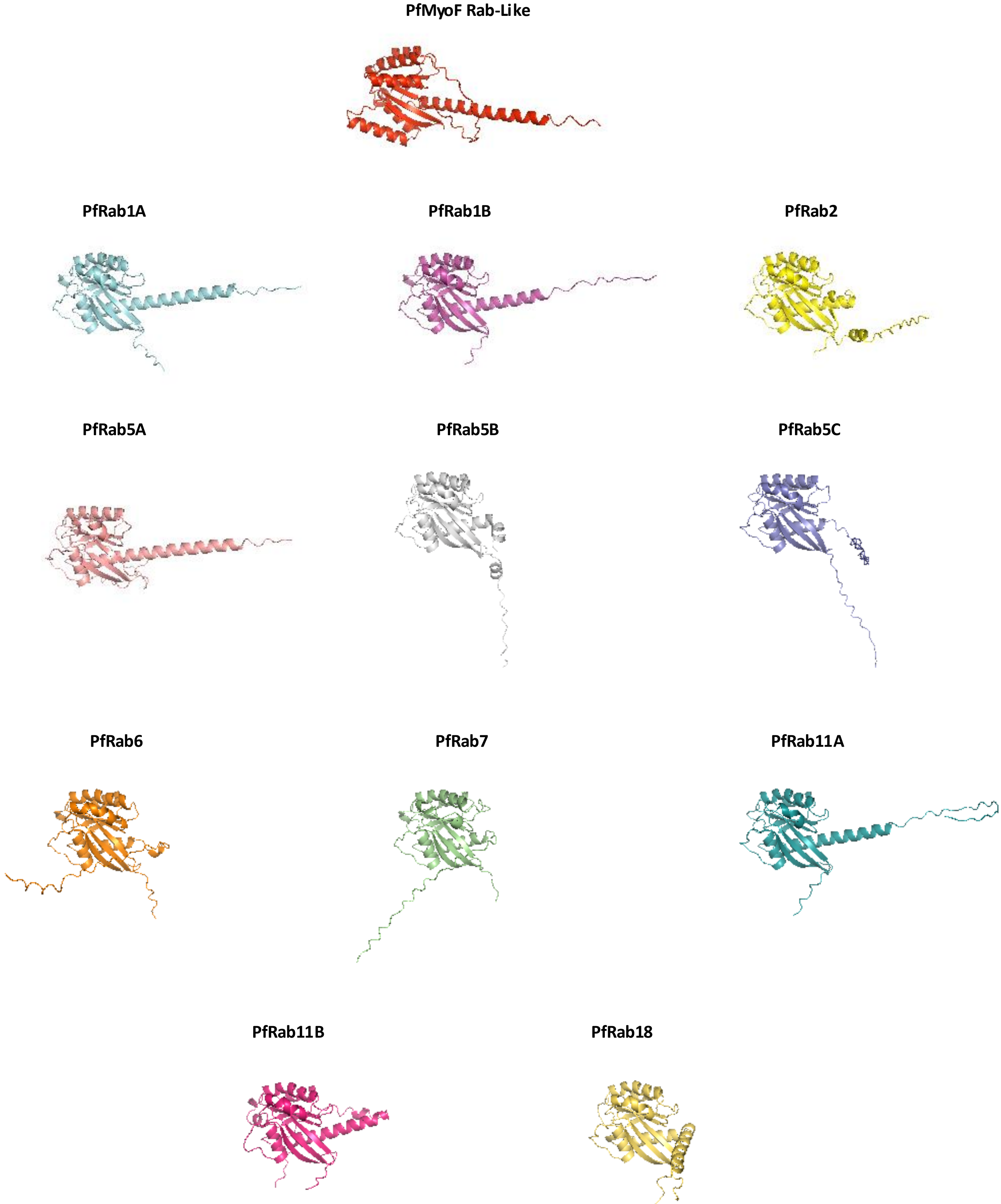

AlphaFold-3 predictions of the PfMyoF Rab-Like domain alongside the 11 Rab proteins of *P. falciparum*, revealing high structural similarity.

Supplementary Figure 4 . Multiple Sequence Alignment and AlphaFold-3 prediction of ‘Insert-2’ regions.

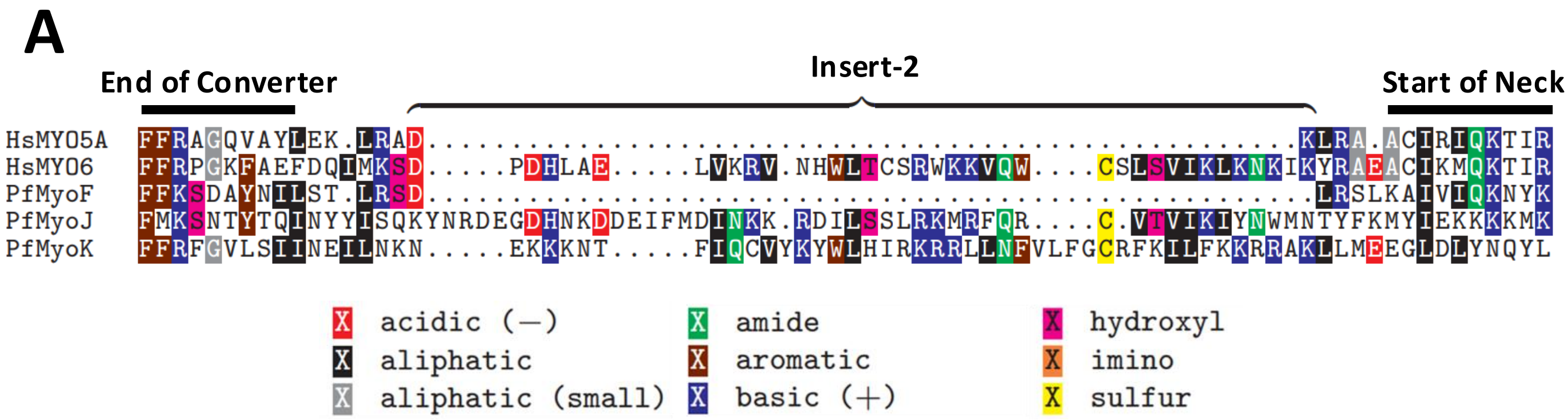

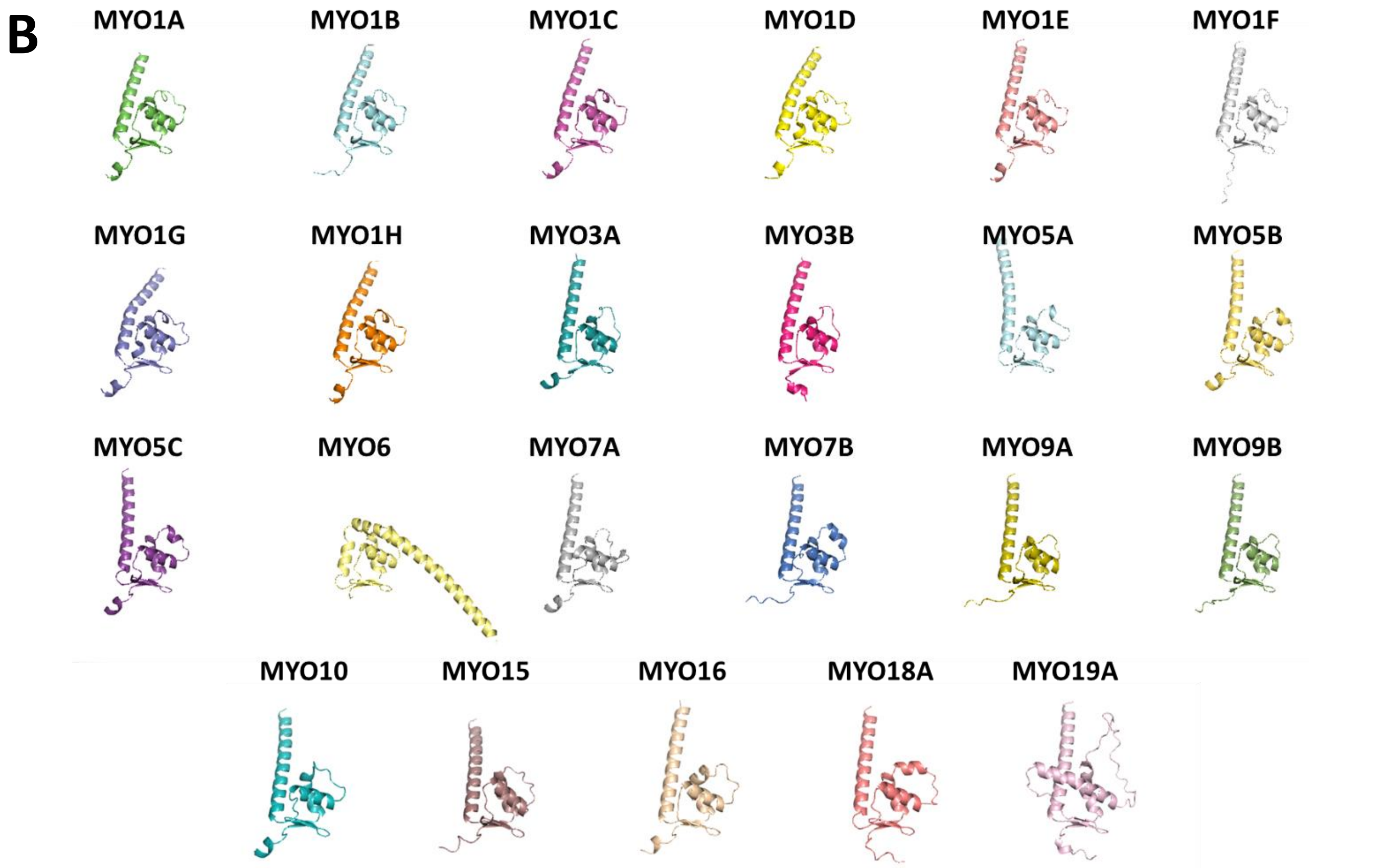

(A) Multiple Sequence Alignment of ‘Insert-2’ regions at the end of the converter domains of HsMYO5A, HsMYO6, PfMyoF, PfMyoJ and PfMyoK. Both HsMYO5A and PfMyoF lack Insert-2, which is present in PfMyoJ and PfMyoK. PfMyoJ contains a sequence strong with strong homology to the Insert-2 of HsMYO6. (B) AlphaFold-3 prediction and alignment of the converter-neck angles of all unconventional human myosin proteins, which identifies HsMYO6 as a minus-end directed motor based on lever arm position.

Supplementary Figure 5.The Archetypes of Filopodia Localisation.

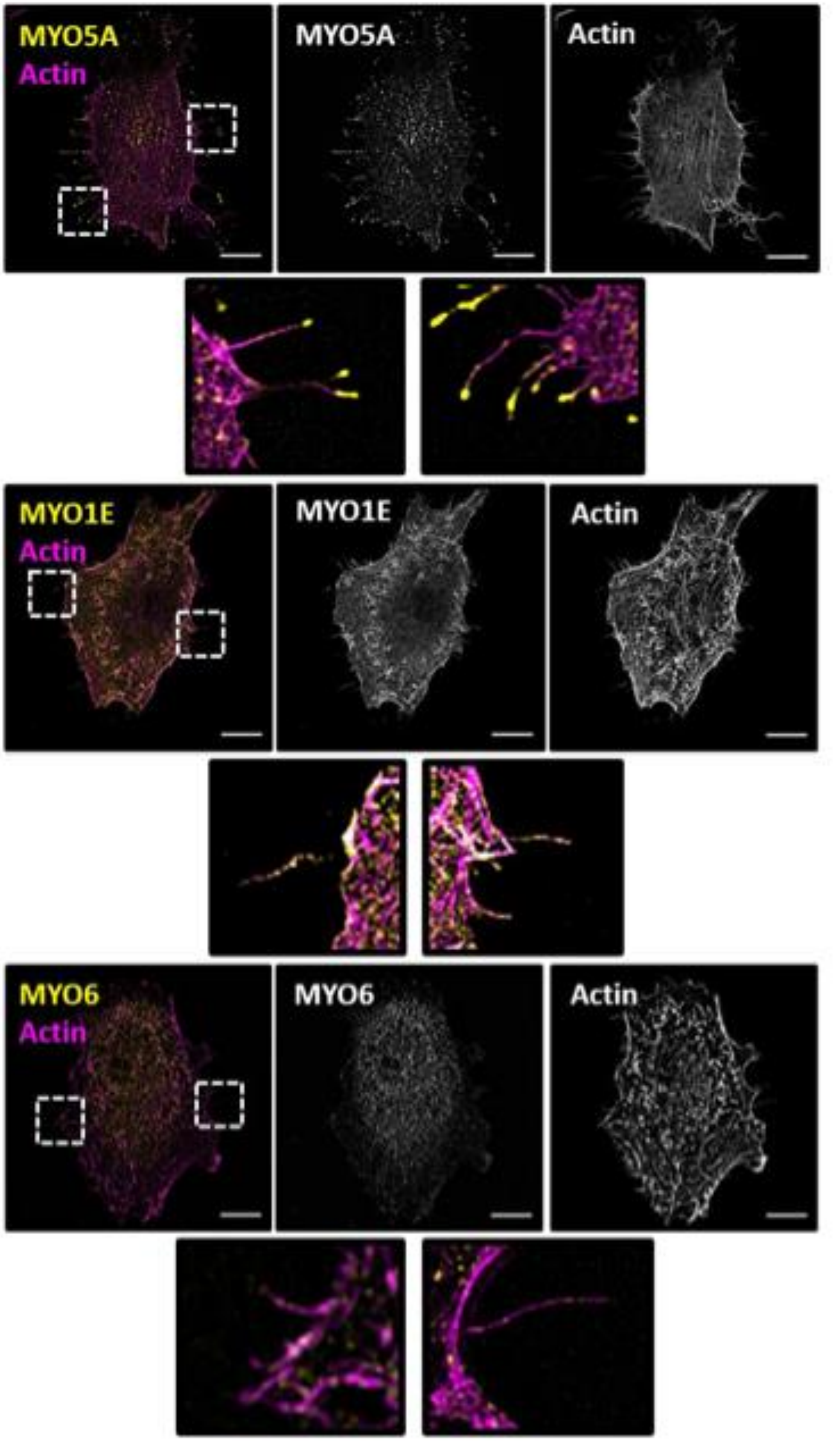

Representative images of three types of myosin localisation in filopodia of RPE cells. Tip end localisation (MYO5A), shaft localisation (MYO1E) and absent (MYO6). Actin (magenta) and GFP-tagged myosin (yellow) are shown. Scale bar, 10 µm.

Supplementary Figure 6. Reactivity of the PfMyoF antibody PreImmune Serum tested on Blood-stage Trophozoites and Schizontes.

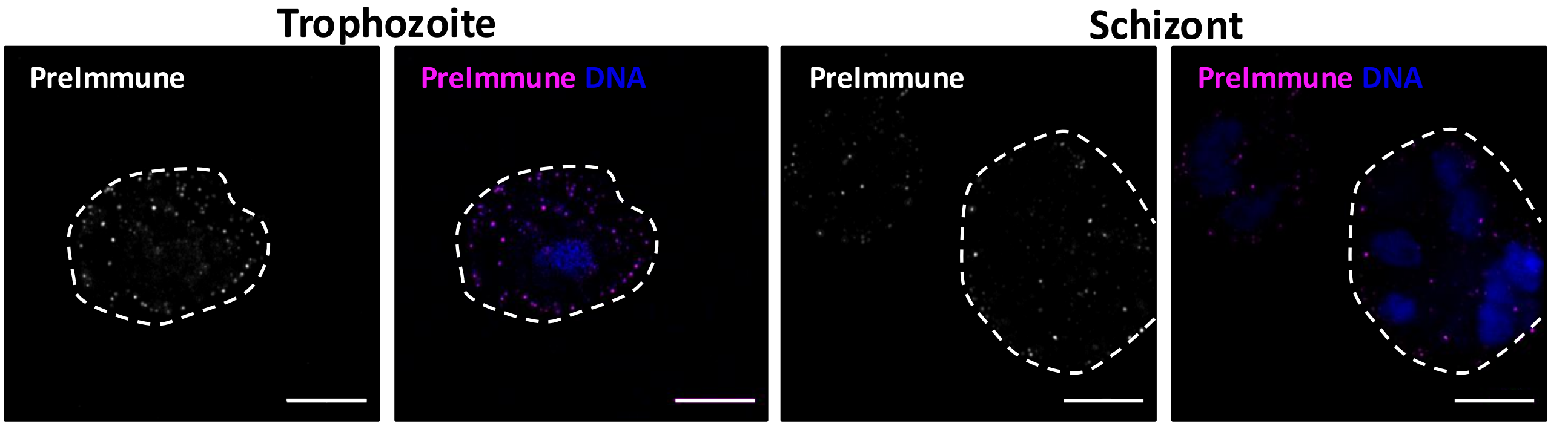

U-ExM of trophozoite (left two panels) and schizont (right two panels) stage parasites stained with the preImmune serum (magenta) and Hoechst (blue). All scale bars represent 10 µm.

Supplementary Figure 7. Genotyping of PfMyoF HA*glmS* transfectants

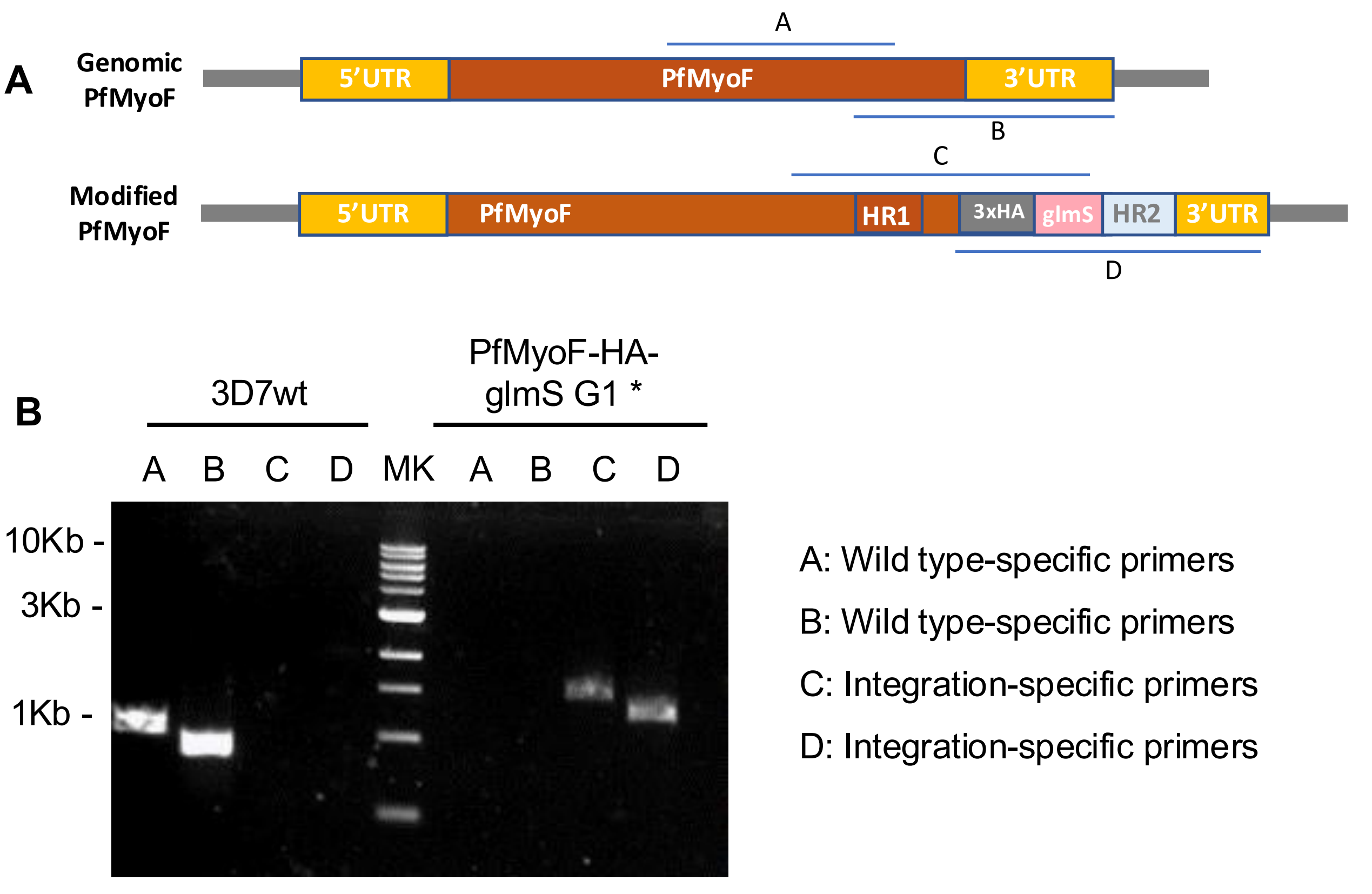

A. Schematic showing the PfMyoF-HA*glmS* tagged construct with genotyping primers and their binding sites. B. DNA agarose gel of genotyping PCR for PfMyoF-HA*glmS* transgenic and 3D7 wildtype lines. HR1 and HR2 represent the flanking homology regions. G1* represents parasite clones carried forward for the downstream analysis.

Supplementary Figure 8. Inducible knockdown of PfMyoF in asexual stage parasites.

A

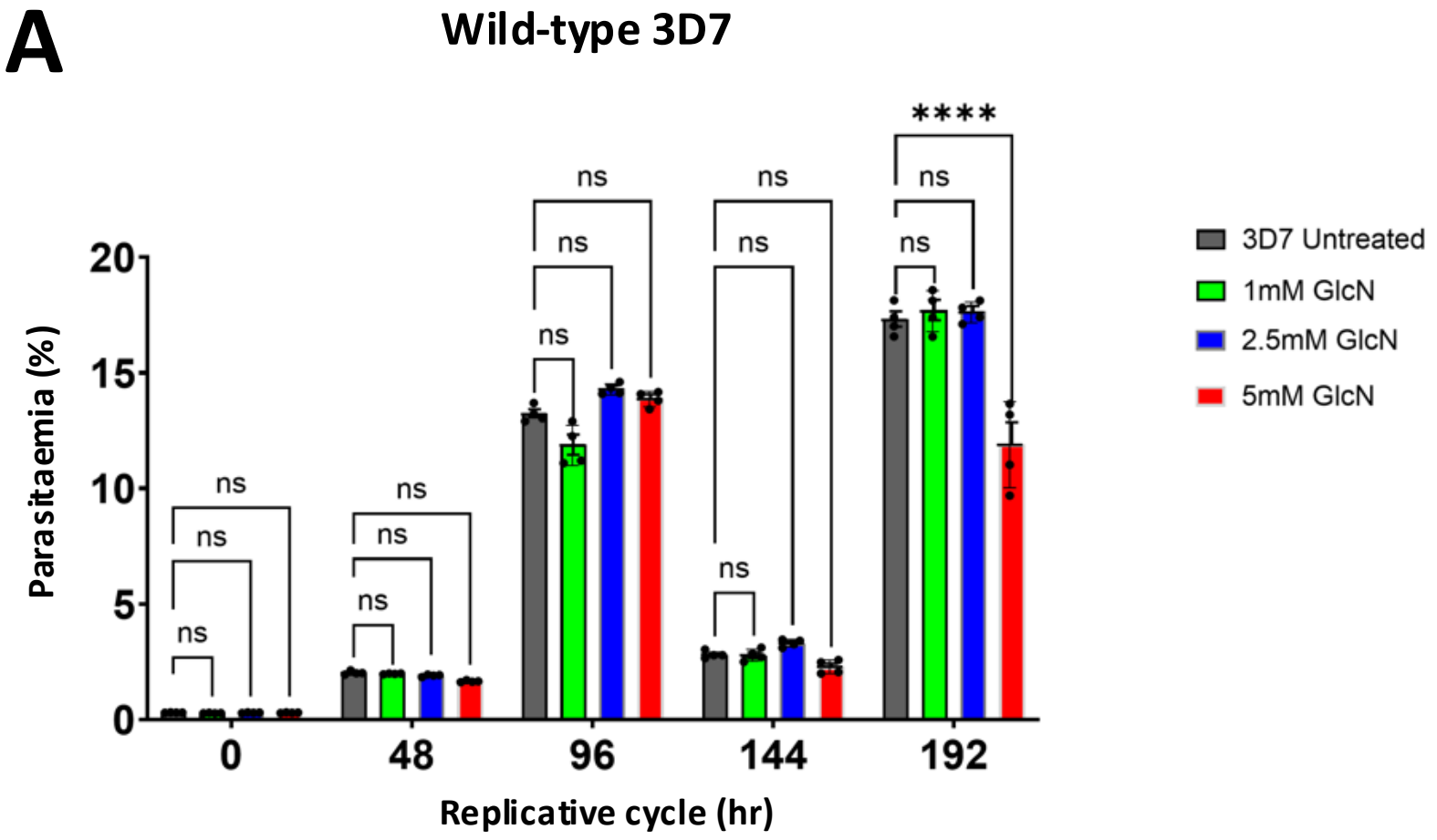

B

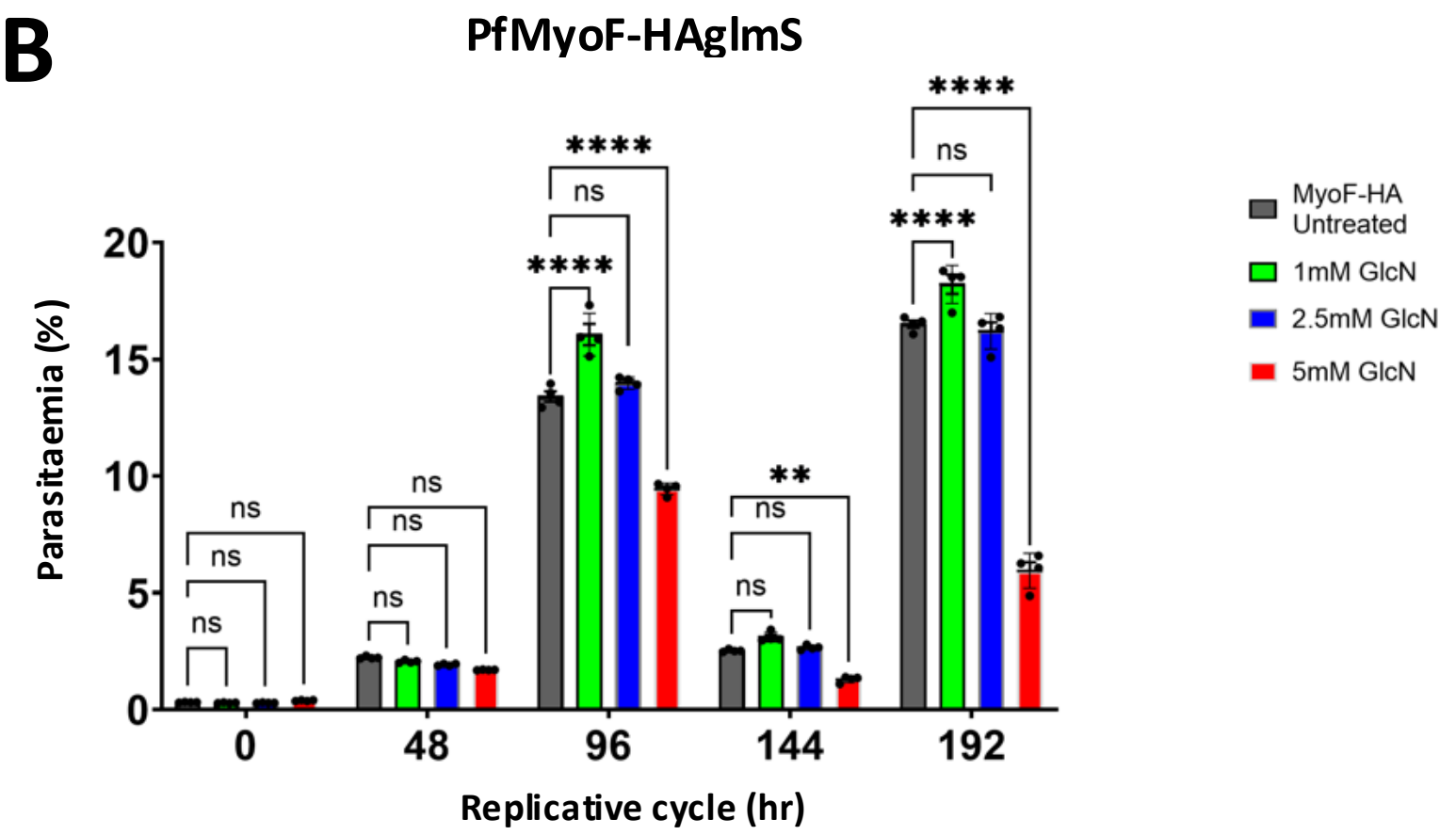

(A and B) Growth analysis of wild-type 3D7 and PfMyoF-HAglmS parasites over four replication cycles. Untreated parasites for each line were compared with their respective parasites grown in the presence of 1 mM, 2.5 mM or 5 mM GlcN. After 96 hours, parasites were diluted 1:40 to prevent parasites from overgrowing and allow monitoring parasitaemia up to 4 replicative cycles. Data are mean ± SEM for n=4 with 3 replicates each. n= 4. GlcN, glucosamine.

Supplementary Figure 9. Uncropped blot for Figure 1H.

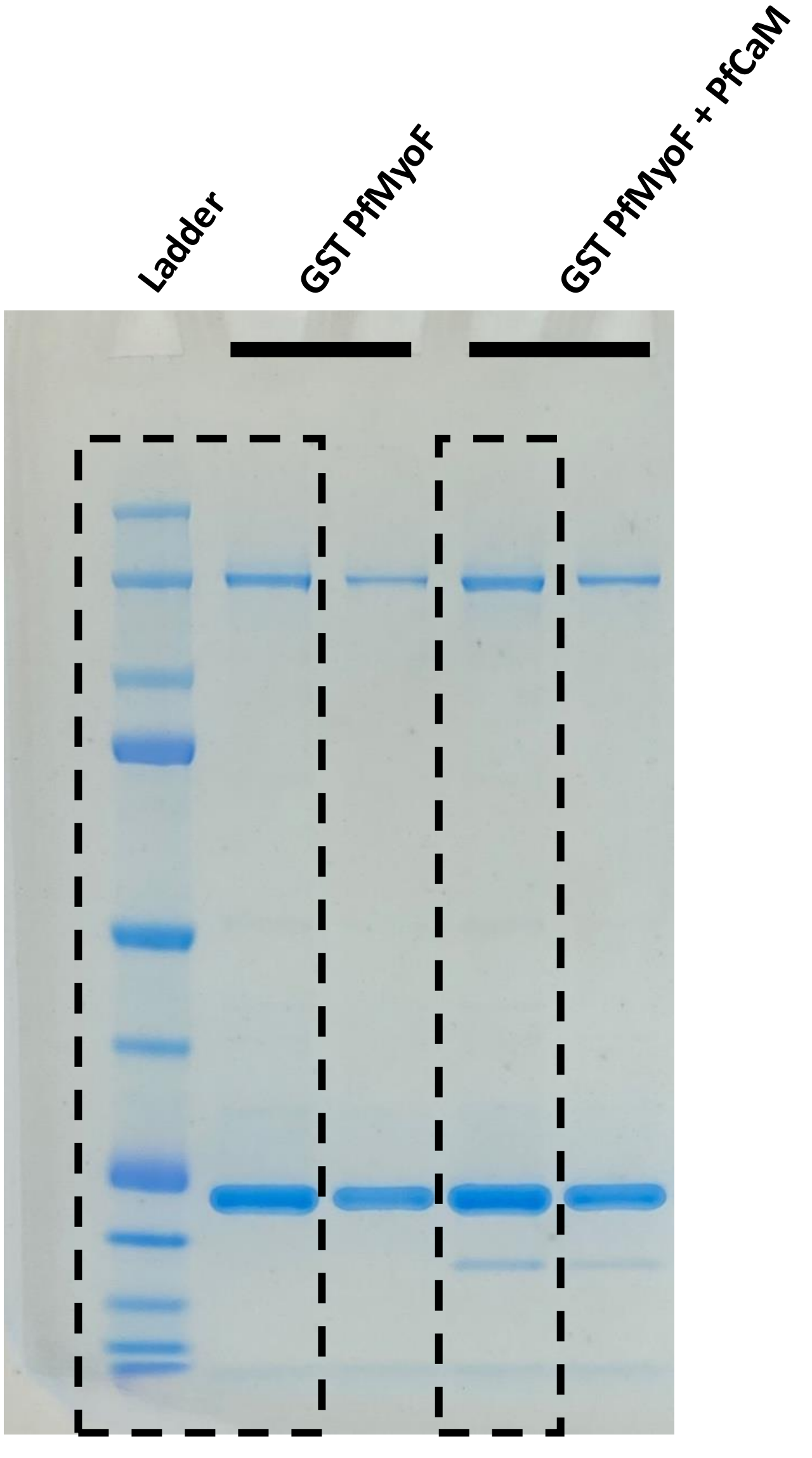

Lanes featuring within Figure 1H are denoted by a dotted line.

Supplementary Figure 10. Uncropped blot for Figure 5A.

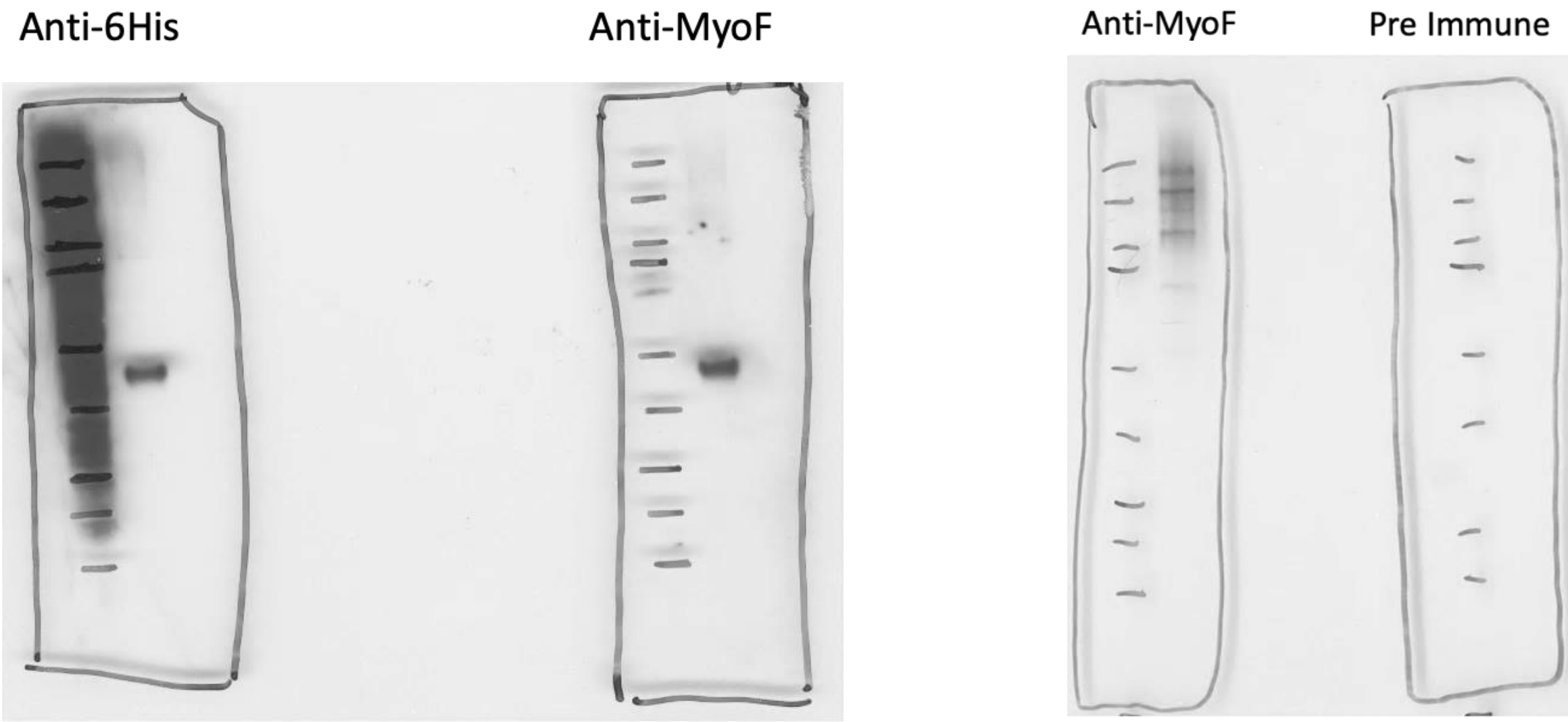

6His-WD40 domain expressed in insect cells

Plasmodium lysate

Supplementary Figure 11. Uncropped blot for Figure 7A.

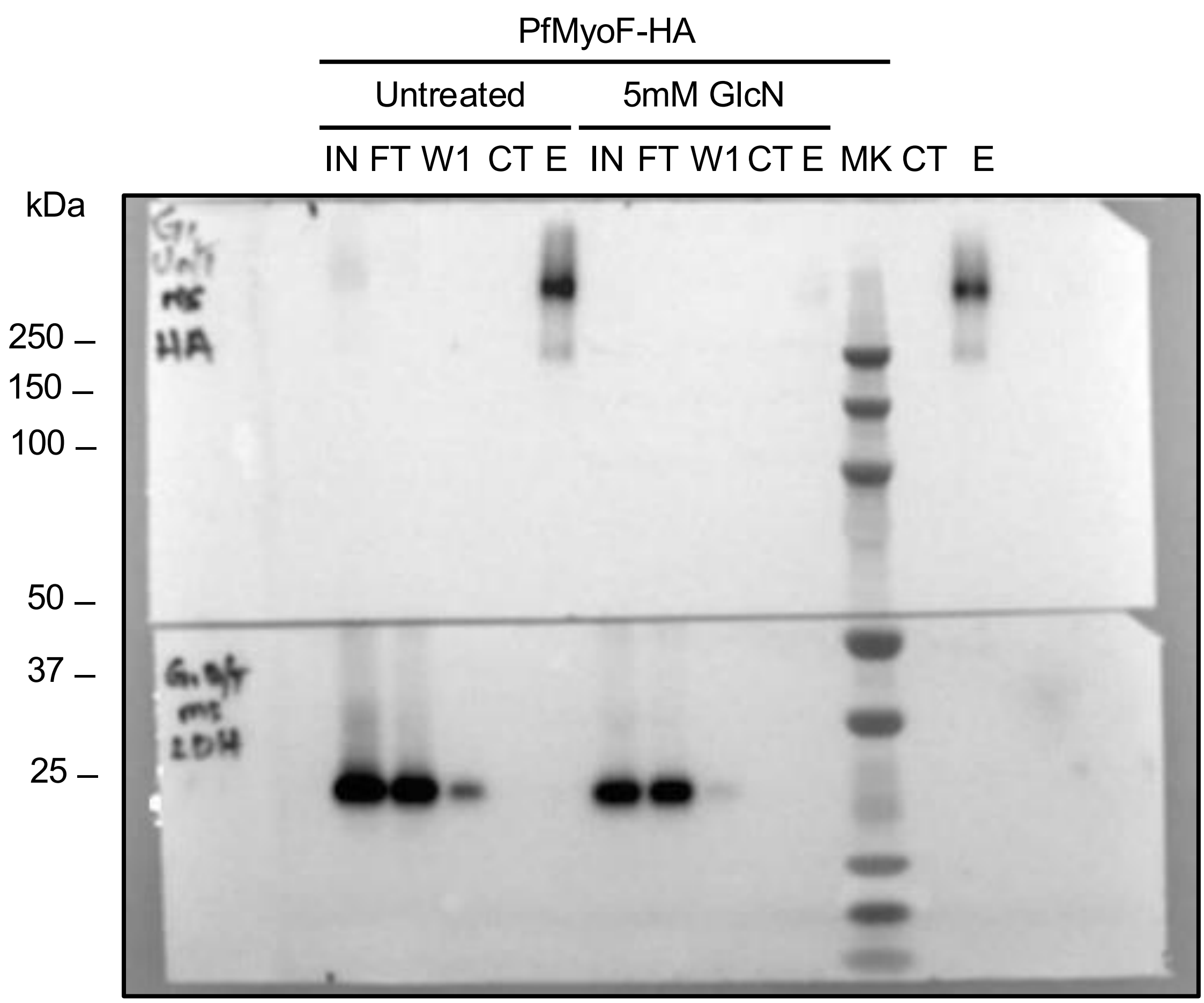

Supplementary Figure 12.
Unedited Image of Agarose Gel shown in Supplementary Figure 7B

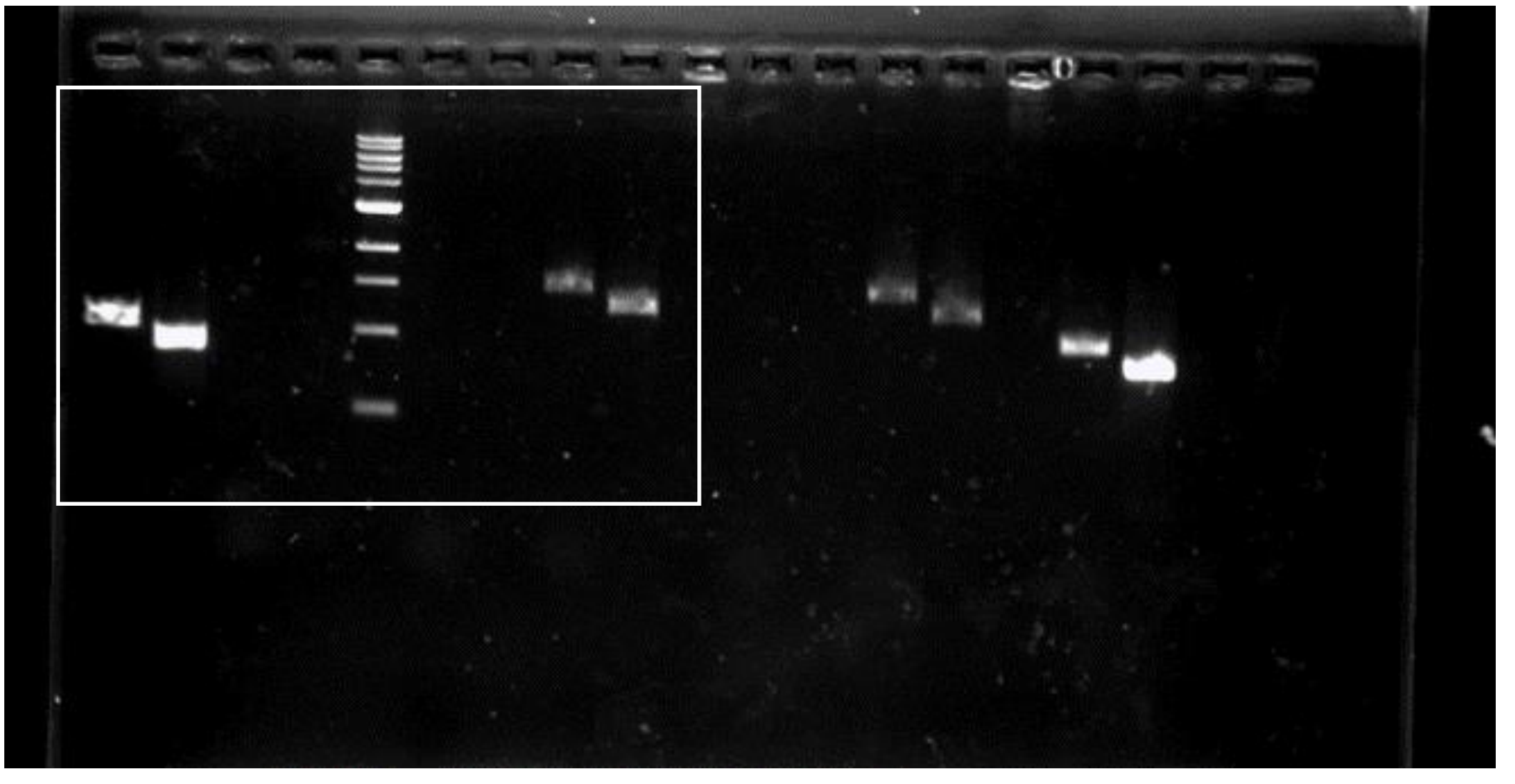

Lanes featuring within Supplementary Figure 7B are highlighted by a white box.
