# Supplementary material for: Plasmodium falciparum PfMyoF contains a Rab-like tail domain and associates with perinuclear membrane trafficking proteins
Source: Commun Biol. 2026 May 15;9:1056. doi: 10.1038/s42003-026-10192-1 (PMC13448217; doi:10.1038/s42003-026-10192-1)
Supplement: Supplementary file 2 [file 42003_2026_10192_MOESM2_ESM.pdf]

Description of Additional Supplementary Files

File name: Supplementary Data 1

Description: The numerical source data for Figure 4E, Figure 5D and Figure 7B
